# Characterization of plasma cytokine response to intraperitoneally administered LPS & subdiaphragmatic branch vagus nerve stimulation in rat model

**DOI:** 10.1371/journal.pone.0214317

**Published:** 2019-03-28

**Authors:** Jesse P. Somann, Kelsey M. Wasilczuk, Kaitlyn V. Neihouser, Jennifer Sturgis, Gabriel O. Albors, J. Paul Robinson, Terry L. Powley, Pedro P. Irazoqui

**Affiliations:** 1 Department of Electrical and Computer Engineering, Purdue University, West Lafayette, Indiana, United States of America; 2 Center for Implantable Devices (CID), Purdue University, West Lafayette, Indiana, United States of America; 3 Weldon School of Biomedical Engineering, Purdue University, West Lafayette, Indiana, United States of America; 4 Purdue Cytometry Laboratories, Purdue University, West Lafayette, Indiana, United States of America; 5 Department of Psychological Sciences, Purdue University, West Lafayette, Indiana, United States of America; University of California Los Angeles, UNITED STATES

## Abstract

Vagus nerve stimulation (VNS) has been on the forefront of inflammatory disorder research and has yielded many promising results. Questions remain, however, about the biological mechanisms of such treatments and the inconsistencies in the methods used in research efforts. Here, we aimed to clarify the inflammatory response to intraperitoneal (IP) injections of lipopolysaccharide (LPS) in rats, while analyzing corresponding effects of electrical stimulation to subdiaphragmatic branches (anterior gastric, accessory celiac, and hepatic) of the left vagus nerve. We accomplished an in-depth characterization of the time-varying cytokine cascade response in the serum of 58 rats to an acute IP LPS challenge over a 330-minute period by utilizing curve-fitting and starting point-alignment methods. We then explored the post-LPS neuromodulation effects of electrically stimulating individually cuffed subdiaphragmatic branches. Through our analysis, we found there to be a consistent order of IP LPS cytokine response (IL-10, TNF-α, GM-CSF, IL-17F, IL-6, IL-22, INF-γ). Apart from IL-10, the IP cytokine cascade was more variable in starting time and occurred later than in previously recorded intravenous (IV) challenges. We also found distinct regulatory effects on multiple cytokine levels by each of the three subdiaphragmatic stimulation subsets. While the time-variability of IP LPS use in rats complicates its utility, we have shown it to be a practical, arguably more physiologically relevant method than IV in rats when our methods are used. More importantly, we have shown that selective subdiaphragmatic neurostimulation can be utilized to selectively induce specific effects on inflammation in the body.

## Introduction

### Background and motivation

There is no shortage of ongoing research revolving around the topic of inflammatory diseases and disorders. Inflammation and related treatments have been linked to disorders including rheumatoid arthritis [1], Crohn’s disease [2], chronic depression [3], and many other ailments. Among currently proposed treatment methods for excess inflammation of the gastrointestinal (GI) tract is that of vagus nerve stimulation (VNS). VNS is currently FDA approved and utilized for epilepsy and drug-resistant depression treatments [4, 5], yet many specific physiological mechanisms in response to VNS are still unclear. In addition, the research community is plagued by inconsistency in experimental methods, making it difficult to replicate results and translate them into future research and treatments.

Central to many inflammation studies is the use of an inflammatory challenge, most commonly using lipopolysaccharide (LPS) endotoxin, to induce an acute inflammatory response that can then be treated by a given drug or therapy. However, the LPS injection method, dosage, and timing can vary greatly between studies, and the results of said experiments may indeed depend in part on these variables. LPS administered through either intraperitoneal (IP) or intravenous (IV) injection is generally thought to produce similar inflammatory responses [6–8], but shows some differences, likely due to distinct mechanisms in the creation of cytokines.

Steven et al. [9] recently made a strong case for better methods characterization and standardization by illustrating differences between LPS challenges in mice and rats. In addition, the dosage of LPS elicits different cytokine creation and timing in their responses [10]. We submit that one important and under-characterized aspect in inflammatory research is the LPS administration method. In VNS research related to inflammation, we most commonly find IV injections used on rat subjects [11–14], while IP injection methods are reserved for studies on mice [15, 16], presumably owing to the technical difficulty of IV insertion into their smaller blood vessels. Studies by Lenczowski et al. [17, 18] demonstrated high variability in response to IP LPS usage in rats; in discussion with other researches in this area, we found such variability to be the main motivation to administer LPS IV in this animal model. We assert, however, that IP administration presents a more physiologically relevant inflammatory simulation. IP application represents a foreign infection to which the body must respond in a more systematic way, whereas IV delivery artificially adds LPS to the blood stream. Therefore, developing methods to better utilize IP LPS injections and a better understanding of the expected responses will benefit a large community of researchers.

In addition to pursuing a better understanding of the effects of the methods we use in inflammation research, we must continue working to narrow in on physiological mechanisms that can be manipulated to treat specific inflammatory responses. Prior work has postulated that there exists an inflammatory reflex by which the nervous system regulates immune function. By stimulating the efferent fibers of the vagus nerve, splenic macrophage release could be controlled, thus regulating cytokine release. In this way, the inflammatory reflex could be modified to produce an anti-inflammatory response [19]. However, the concept of direct innervation of the spleen by the vagus nerve was met with uncertainty as early as 1993 [20]. More recent research has proposed that vagal innervation of the celiac ganglia is the first in a two-part relay, in which vagal action potentials regulate T cell production, thus controlling the production of the neurotransmitter acetylcholine [16]. A subsequent study showed that VNS inhibited splenic nerve activity but produced no response when the nerve was severed cranial to the stimulation site. This implies that the link between the vagus and spleen is an indirect connection through the central nervous system (CNS) rather than a direct pathway [13].

Along with others, we suggest that the GI vagal innervation proposed by Berthoud et al. in 1991 [21] modulates the release of acetylcholine-synthesizing T-lymphocytes in the blood. By stimulating the vagus, distal gastrointestinal fibers stimulate lymphoid tissue into releasing cells such as these acetylcholine-synthesizing T-lymphocytes. When taken up by the spleen, these lymphocytes cause splenic macrophages to decrease their production of inflammatory cytokines, leading to the desired therapeutic effect [22].

As the left cervical vagus nerve passes through the diaphragm of the body, it becomes the anterior vagal trunk and splits into multiple branches [23]. Previous work by multiple groups has shown left cervical vagus stimulation to attenuate cytokine levels [11, 12, 14, 24] and a recent study by Komegae et al. looked at stimulation of the subdiaphragmatic trunk [25]. In addition, Stakenborg et al. [26] presented effects of VNS on both the anterior and posterior subdiaphragmatic nerves in mice. None of those studies, however, analyzed the effects of stimulation of each individual subdiaphragmatic branch. Berthoud and Powley have previously postulated that vagal celiac branches contribute the most toward innervation of the major ganglia that comprise the solar plexus, followed to a lesser extent by the gastric and hepatic branches [27, 28]. Following this rationale, we hypothesized that stimulation of different isolated subdiaphragmatic branches would mediate inflammation in unique ways. If true, then utilization of vagal branch stimulation to regulate circulating cytokines produced by splenic macrophages could allow selective symptoms of multiple inflammatory diseases to be alleviated, leading to new potential treatment options.

### Experimental objectives

Our preliminary study had three primary objectives. 1) To develop methods for effective analysis of the cytokine cascades caused by an IP-administered LPS challenge. We accomplished this by testing and utilizing a curve-fitting and alignment method on temporal cytokine profiles. 2) To use these methods to more fully characterize the time-varying effects over a 5.5-hour period of IP-administered LPS on multiple systemic cytokine levels and the relationships between those cytokines. In doing so, we were able to map the order and timing of seven cytokine responses. 3) To apply the first two objectives to explore inflammatory effects of electrical neuromodulation directly to the subdiaphragmatic anterior gastric branch (AGB), accessory celiac branch (ACB), and hepatic branch (HB) of the left vagus nerve. We tested 58 total rats for verification and preliminary analysis purposes and discovered distinct sets of modulatory trends caused by each branch.

## Methods

### Animals

The Purdue Animal Care and Use Committee (PACUC) approved all protocols in this study (protocol number 1508001286A009). We used 58 adult male Sprague-Dawley rats (Envigo; Indianapolis, IN) weighing between 175 and 265 g. Animals were housed under standard conditions with ad-libitum access to food and water, unless otherwise stated.

### Electrical stimulation experiments

#### Animal groups

We established two primary surgical groups–the first to study effects of stimulation of anterior subdiaphragmatic branches of the left vagus nerve and the second to study comparative effects of left cervical VNS. Rats undergoing subdiaphragmatic cuffing were separated into sub-groups as illustrated in Table 1. For each anterior branch (anterior gastric, accessory celiac, hepatic), animals received either an LPS challenge with no stimulation, LPS with electrical stimulation treatment, or LPS with electrical stimulation and an efferent vagotomy. An efferent vagotomy was included, both to supply us with clues on the effects of efferent versus afferent stimulation and to simulate the effects of potential damage from nerve cuffing that we have previously reported [29]. Afferent vagotomy subsets were not done because of limited resources for this preliminary study. The final group consisted of saline controls (n = 3), one animal per anterior branch, that were cuffed but received saline in lieu of LPS and no stimulation. For statistical purposes, the subdiaphragmatic non-stimulation subsets (SubD Sham) were grouped. Surgeries were randomized between sub-groups.

**Table 1:** Overview of subgroups used for acute electrical stimulation experiment.

Stimulation Cuff Location	LPS & No Stimulation		LPS & Stimulation		LPS, Stimulation, & Efferent Vagotomy	
Left Cervical Vagus Nerve	CVns	n = 3	CVes	n = 5	N/A	N/A
Anterior Gastric Branch	SubD Sham	n = 8	AGBes	n = 5	AGBvx	n = 5
Accessory Celiac Branch			ACBes	n = 3	ACBvx	n = 4
Hepatic Branch			HBes	n = 4	HBvx	n = 5

The cervical group was divided between animals that received an LPS challenge and a 5-minute stimulation treatment and those that received LPS but did not receive stimulation. In addition, we performed a sub-set of validation animals (n = 4) to verify our curve-fitting methods.

#### Materials

We fabricated custom bi-polar stimulation cuffs (S1 Fig) using platinum-iridium wire and medical-grade silicone tubing, as done previously [29]. The cervical cuffs used in this study have been previously characterized [30]. Our cervical electrodes had a measured impedance of 1.40–1.62 kΩ at 1 kHz. The subdiaphragmatic cuff electrodes had a 0.02” inner diameter, but no suture, and an impedance of 2.07–3.45 kΩ at 1 kHz.

An improved version of the Bionode stimulator used previously [29] was utilized for our acute stimulation experiments. The stimulator output charge-balanced, bi-phasic, square wave pulses of up to 1.1 mA constant current amplitude. To prevent stray current from permeating the animal, we added a direct current (DC)-blocking capacitor in series with the stimulator and included circuitry to short the capacitor between stimulation pulses to prevent charge build-up.

#### Acute surgical procedures

We anesthetized rats using 4–5% isoflurane for approximately 3 minutes, then weighed and injected the animals IP with a ketamine/xylazine cocktail consisting of 75 mg/kg ketamine and 5 mg/kg xylazine. A supplement of buprenorphine was administered subcutaneously (0.08–0.12 mg/kg). Anesthesia was maintained with IP injections of ketamine/xylazine every 30 minutes or as needed based on heart rate and toe pinch. The combination of anesthetics and analgesia used was determined to have a minimal effect on inflammatory cytokine variations, based on previous literature [31]. We shaved and cleaned the incision sites and applied artificial tears to the eyes. The animal was then placed supine on a heating pad and placed under oxygen flowing at 2 L/min for the remainder of acute surgery and blood collections. We euthanized animals at experiment completion via a lethal dose of Beuthanasia-D Special (Merck; Kenilworth, NJ) (0.9 mL, IP).

Catheters for blood collection were placed before cuffing the vagus nerve branch. We followed the general procedures outline in the Culex surgical manual (Bioanalytical Systems, Inc.; West Lafayette, IN) for femoral cannulation in a rat [32]. Catheters in our experiment, however, were inserted into the femoral artery instead of the vein and remained exposed on the benchtop for connection to the Culex system versus being subcutaneously routed through the animal.

For animals in the cervical cuffing group, we made the initial incision at the midline of the jaw, moving caudally. We exposed soft tissue until the sternohyoid and sternocleidomastoid muscles sitting on top of the carotid sheath were visible. Via blunt dissection, we gently separated connective tissues until the carotid artery sheath could be seen. The carotid sheath was carefully dissected until the left vagus nerve was completely free from surrounding tissues. We then placed the cuff electrode under the vagus nerve, slid the nerve into the cuff, and tied the attached suture to secure the cuff in place. The area was kept moist with saline and covered with gauze for the duration of the surgery when stimulation was not being applied.

Animals in the subdiaphragmatic cuffing group were fasted for 6–12 hours before surgery to reduce stomach volume and improve access to the subdiaphragmatic nerve branches. To expose the subdiaphragmatic branches of the vagus nerve we made an incision starting at the midline above the xyphoid process and moved diagonally to the animal’s left. The muscle was blunt dissected along the incision and held open using an elastic retraction system. We carefully retracted the left liver lobule, cut the hepatoduodenal ligament, and moved the right liver lobule aside to reveal the esophagus. We then located and isolated either the anterior gastric, accessory celiac, or hepatic branch of the left vagus nerve and carefully attached a subdiaphragmatic cuff electrode. The incision site was left open through electrical stimulation therapy to ensure that the cuff remained secure on the nerve. For animals receiving an efferent vagotomy, we tied off the nerve branch with 6–0 silk suture, caudal to the cuff, and cauterized the nerve fibers caudal to the suture tie. The suture physically prevented the retraction of the nerve after cauterization and kept the cuff from detaching from the nerve during stimulation.

#### Acute blood collection protocol

Prior to catheter placement, we primed the Culex Automatic Blood Collection System (Bioanalytical Systems, Inc.; West Lafayette, IN) [33] with heparinized saline. Once the catheter was placed in the animal, we connected it to the Culex for tending during the remaining surgical procedure.

**Fig 1:**
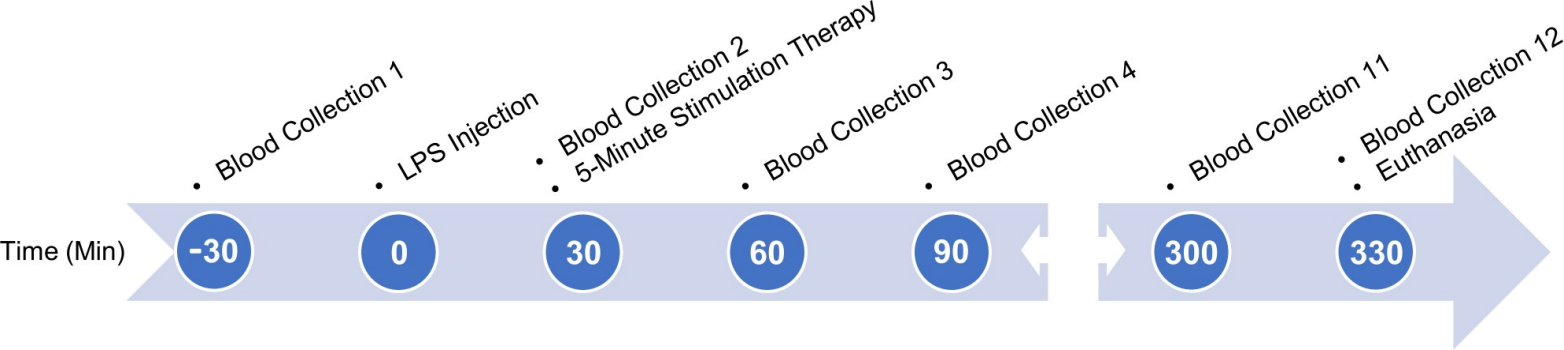
Time flow diagram outlining the experimental process. Process included blood collection, LPS administration, and stimulation treatments (if applicable). Time points are based in relation to the acute LPS challenge to the animal.

Our blood collection protocols followed those outlined in the NIH Guidelines for Survival Bleeding of Mice and Rats. We programmed the Culex system to automatically collect 12 separate 115-μL blood samples, spaced 30 minutes apart after the second collection, over a period of six hours as outlined in Fig 1. An initial baseline collection was done 30 minutes prior to LPS injection. In doing so, we did not exceed the maximum allowed 10% of circulating blood volume. Blood was collected into vials containing K_3_EDTA (1.75 mg/ml) and centrifuged at 2000× g for eight minutes after every two collections. We then extracted the plasma, aliquoted it into 20-μL samples, and stored them at -20°C until they could be processed.

Validation animals were treated in a similar fashion. Blood collections were doubled in number (24 samples) but halved in volume to compensate. Saline was given periodically throughout the procedure to replenish fluids lost with increased rate of blood collection.

#### LPS preparation/administration

To ensure proper dosing (5 mg/kg) we prepared all LPS (Sigma-Aldrich, serotype O111:B4) solutions for acute animals in advance. We systematically added 20 mL sterile saline (0.9% sodium chloride), mixed with a new 100 mg bottle of LPS lyophilized powder, and sonicated the mixture for a total of 45 minutes to ensure complete dissolution. We then aliquoted ~400 μL solution into individual 1.5 mL Eppendorf tubes, before freezing and storing at -20°C until used.

Individual LPS aliquots were thawed by sonication for 30+ minutes at room temperature 40–120 minutes before use. We then sonicated the aliquots for an additional 5 minutes directly before administering the IP LPS challenge to the animal at the specified time during our protocol.

#### Stimulation

Stimulation, when applicable, was performed 30 minutes after injecting LPS, following completion of the second blood collection. The stimulator was connected to the surgically affixed stimulation cuff, in parallel with an oscilloscope for recording pulses, and was powered on promptly before stimulation therapy.

We performed all stimulation treatments for 5 minutes with balanced bi-phasic, square wave pulses, but utilized two separate sets of stimulation parameters. For the cervical vagus nerve, we used a larger amplitude profile (1 mA, 200 μs, 5 Hz) that corresponded closely to effective electrical charge used in other literature [12, 14, 16] and showed good action potential response in previous testing. The stimulation intensity was reduced for our subdiaphragmatic stimulation treatments (100 μA, 200 μs, 5 Hz), owing to the reduced size and fiber makeup of the subdiaphragmatic nerve branches. This amplitude was shown to be sufficient to affect the inflammatory response to LPS in a previous study [34].

Prior to each stimulation treatment, we verified the stimulator for consistency and balance using a known resistive load and oscilloscope. During stimulation, we utilized the oscilloscope to verify an active stimulation and to record voltage profiles of the stimulation pulses. Cervical stimulations revealed voltage amplitudes ranging from 3.24 V to 3.93 V, resulting in relative interface impedances of 3.24 kΩ – 3.93 kΩ. Alternately, subdiaphragmatic stimulations showed voltage amplitudes ranging from 0.83 V– 1.40 V, with relative interface impedances of 8.3 kΩ – 14.0 kΩ.

#### Plasma processing

We analyzed plasma samples using a LEGENDplex Rat Th Cytokine Panel (13-plex) (Biolegend, San Diego, CA), which allowed simultaneous analysis of 13 cytokines: Interleukin (IL)-2, IL-4, IL-5, IL-6, IL-9, IL-10, IL-13, IL-17A, IL-17F, IL-22, granulocyte-macrophage colony-stimulating factor (GM-CSF), interferon (IFN)-γ and tumor necrosis factor (TNF)-α. The cytokine kit was modified for use with 384-well plates and the samples processed, as described previously [30]. Many of our IL-6 samples showed levels far above the upper limits of our established flow-cytometry methods. To compensate, we re-ran the saturated samples using a further dilution factor of 50:1.

### Data processing techniques

Owing to limits on amount and timing of blood collections, along with a high temporal variation in cytokine response to IP LPS injections, we pursued simple new techniques that would allow us to better characterize resulting cytokine cascades.

#### Curve fitting

The first method we utilized was that of a smoothing spline interpolation of the 30-minute time points. We ran our 12 recorded time samples through a piecewise polynomial smoothing spline interpolation using Matlab (R2017b, Curve Fitting Toolbox 3.5.6) with a smoothing parameter of p = 1 to ensure that all sample values were preserved. From these interpolations, we pulled new sets of values that could be defined by minute increments and allow a better estimate of peaks, starting points, and areas under the curves. Other interpolation methods such as Gaussian and polynomial fitting, and cubic and shape-preserving interpolants were tested, but the smoothing spline provided the most accurate estimate of actual cytokine curves.

#### Curve alignment

**Fig 2:**
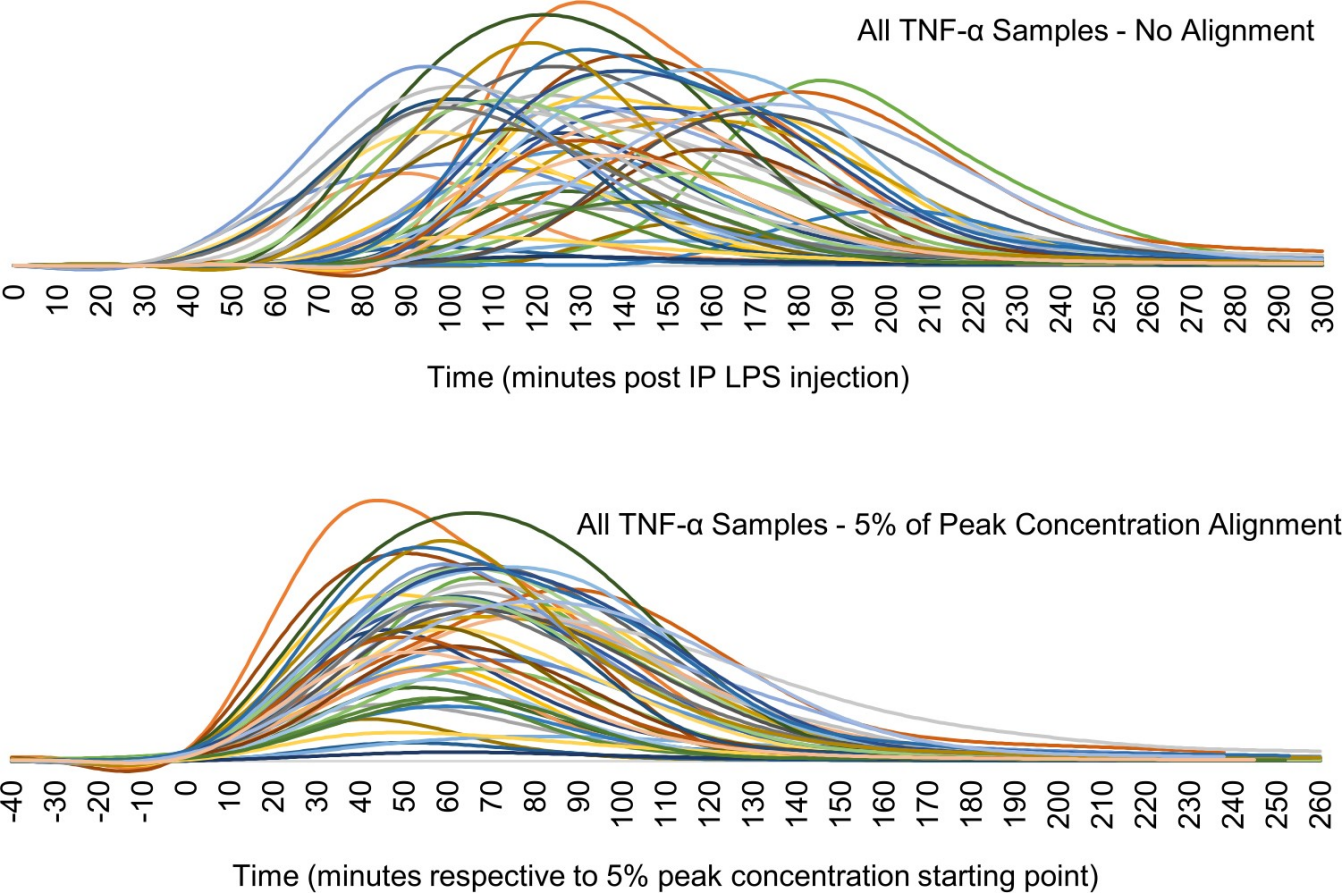
Comparison of individual TNF-α temporal profiles from all rats analyzed with LPS administered IP. Top shows unaligned plots with highly variable starting points. Bottom shows the result of aligning utilizing our technique of 5% peak concentration as a starting point (t = 0).

The second method that we implemented was one of aligning the resulting cytokine curves to a standard point. This is non-trivial, as different cytokines display distinct temporal profiles in amplitude and shape, as well as high animal-to-animal variation. After multiple tests, we settled on using a starting-point alignment, defining the starting point for a given cytokine uptake as the point where it reaches 5% of its peak concentration value (or first peak, if there are multiple). If late-rising cytokines had not yet reached a peak, we utilized the last concentration recorded as the effective peak for alignment purposes. This effectively aligned all curves to a standard point in their respective uptake, while minimizing the significance of varying temporal profiles, as can be seen in Fig 2. By aligning all cytokine samples to the same starting point, we are also able to better analyze temporal variances in cytokine uptake between subsets.

#### Verification of curve fitting and alignment

**Fig 3:**
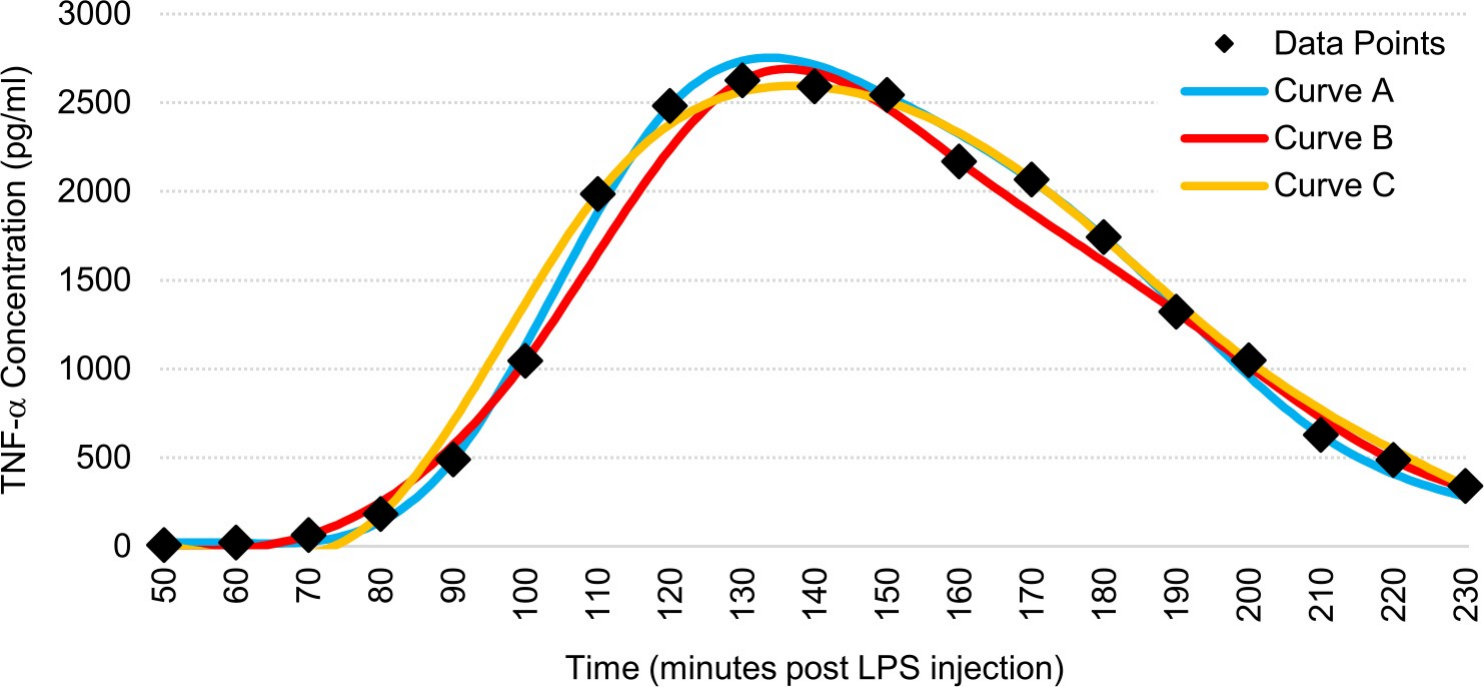
Representative figure demonstrating the effectiveness of our curve-fitting method on a TNF-α cytokine cascade. Data points were taken every 10 minutes. Curve A utilized only points at 60, 90, 120, 150, 180, and 210 minutes. Curve B utilized only points at 70, 100, 130, 160, 190, and 220 minutes. Curve C utilized only points at 50, 80, 110, 140, 170, 200, and 230 minutes.

We ran validation tests on four rats to verify that our interpolation method accurately estimated the actual cytokine cascade curves. For these tests we collected blood in 10- or 15-minute increments for a more accurate estimate of the curve and then split them into 30-minute increment sets as were used for our experimental subgroups. Average variances in calculated starting point between curves were less than three minutes, and average variances in calculated areas under the curve were less than 3%. An example of this validation can be seen in Fig 3 and quantitative results can be found in S1 Table.

### Data and statistical analysis

Data analysis was performed after aligning each cytokine curve based on the 5% of peak concentration method outlined previously. We detected and removed outliers based on interquartile range, or by biological and surgical anomalies, as appropriate, as outlined in the S1 Appendix. Statistical analyses were accomplished using Microsoft Excel Analysis Toolpak and Matlab R2017b statistical functions. One-way ANOVA was performed on the mean calculated starting points of each subgroup. Effects of subdiaphragmatic branch stimulation on cytokine elevations in response to LPS was assessed using one-way ANOVA on mean effective areas under the curves, followed by post-hoc analysis by Student’s t-Test. We considered confidence intervals of 90% and 95% as statistically significant benchmarks for this preliminary study, but we also noted other scientifically significant trends that did not meet these thresholds.

We considered other methods of comparing cytokine profiles between animals and subgroups, such as using a distance matrix with quadratic-form or time-warping distance measures, which could present more robust statistical and quantitative differences between samples. However, we found that these methods did not provide further clarity into the data over those observed when separating the time and concentration variables as we present here. Additionally, separating the variables allowed us to analyze intricacies of actual cytokine uptake profiles in the time domain.

## Results

### Temporal cytokine profiles and relationships

#### LPS-induced cytokine cascade via IP injection

Through a combined subset analysis of recorded cytokine profiles, we detected increased levels in seven out of the 13 cytokines tested during our 330-minute sampling period post IP injection. IL-10, TNF-α, IL-6, and INF-γ had the largest and most consistent elevations, while GM-CSF, IL-22, and IL-17F showed less consistent elevations over the noise floors of our cytometry methods.

**Fig 4:**
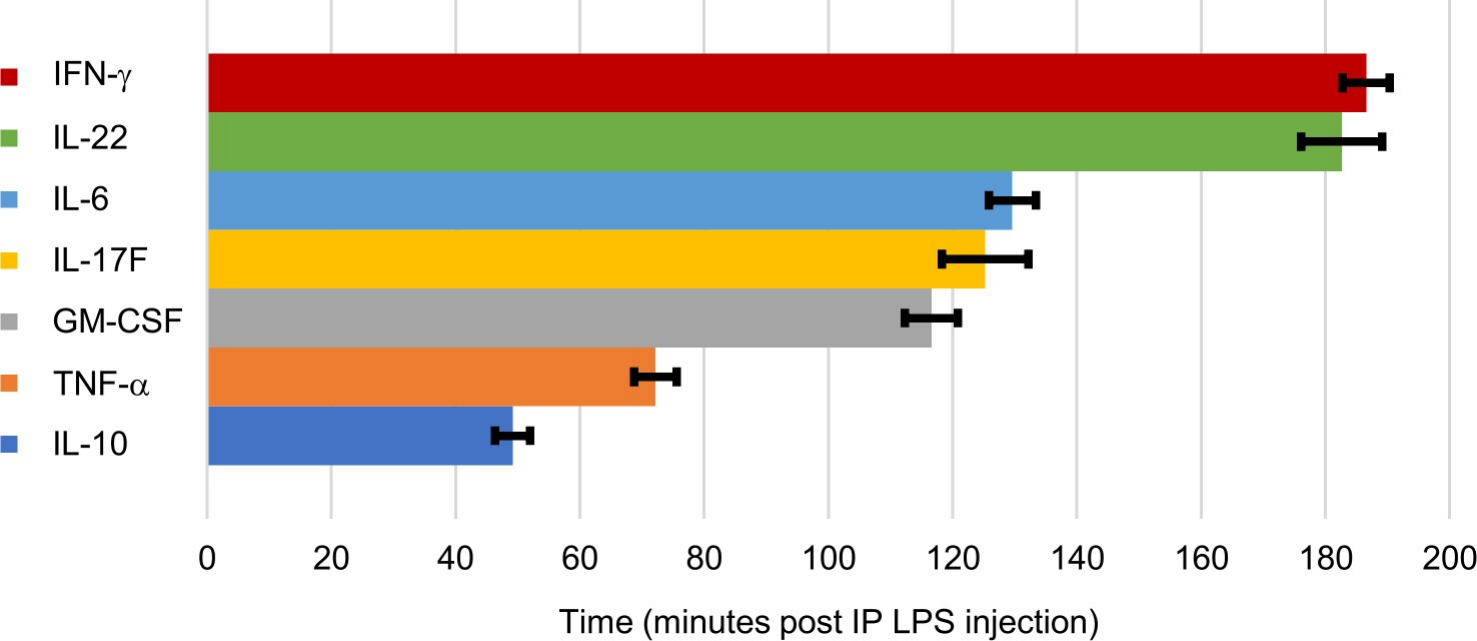
Mean starting times of cytokine elevations. Based on 5% of maximum elevation after IP injection of LPS (5 mg/kg). Error bars, s.e.m.

**Fig 5:**
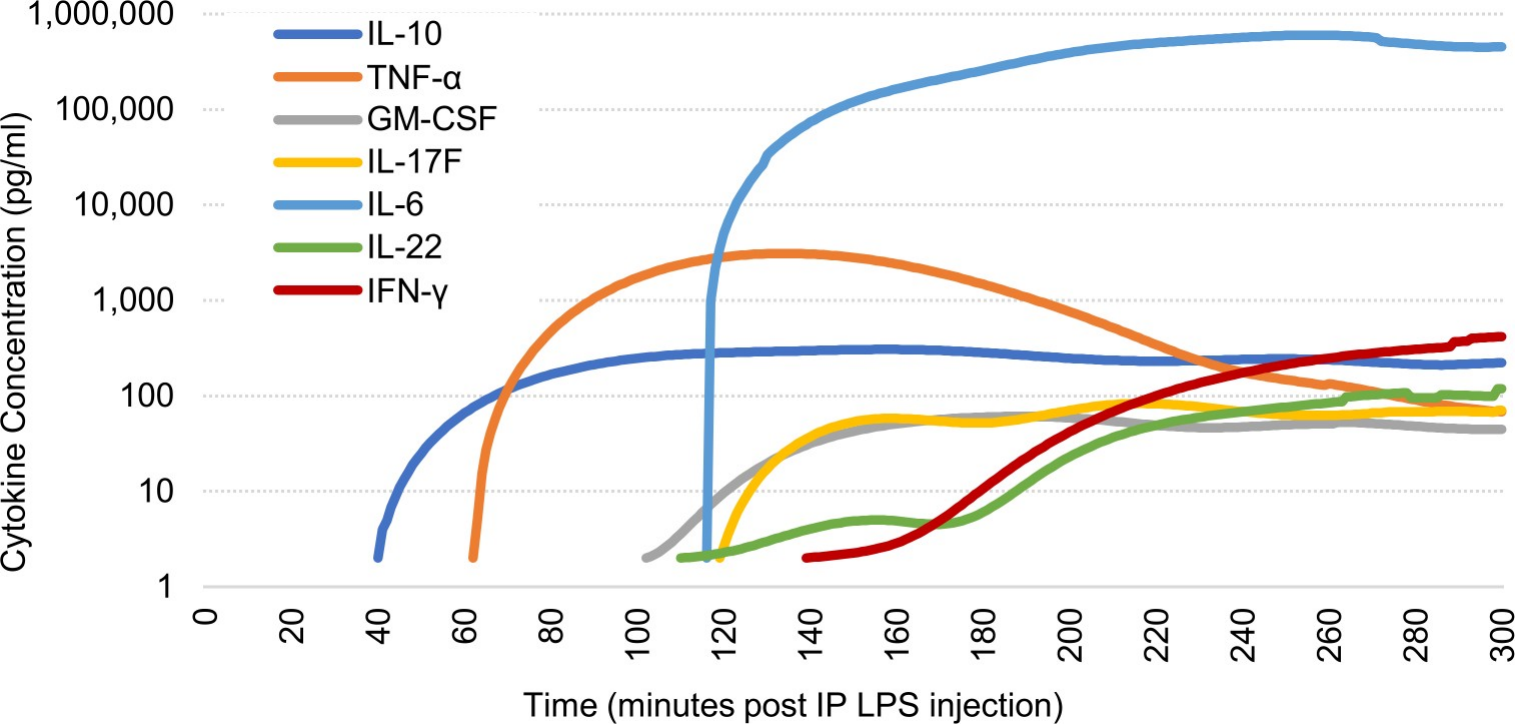
Averaged temporal cytokine profiles of SubD Sham (n = 8) subgroup. Average cytokine cascade due to an IP LPS injection (5 mg/kg) at time t = 0. Logarithmic scale is used to show all elevated cytokines.

Calculated starting times of each cytokine elevation and the mean temporal profiles of our SubD Sham group are illustrated in Figs 4 and 5, respectively. IL-10 is the first cytokine to rise, at 49.2 (± 2.8 s.e.m., n = 44) minutes post-IP LPS injection, followed by TNF-α at 72.1 (± 3.5 s.e.m., n = 44) minutes. We then saw an extended delay before elevations occurred in GM-CSF at 116.6 (± 4.3 s.e.m., n = 44) minutes, IL-17F at 125.2 (± 7.0 s.e.m., n = 29) minutes, and then IL-6 at 129.6 (± 3.8 s.e.m., n = 41) minutes post-LPS. Another hour-long delay then occurred before the elevations of IL-22 at 182.6 (± 6.5 s.e.m., n = 26) minutes and IFN-γ at 186.6 (± 3.8 s.e.m., n = 44) minutes post-LPS injection. No statistical differences in recorded starting points were noted between stimulation and control subsets; therefore, all samples were combined for a more robust temporal characterization.

It is important to note that the observed temporal cascade of cytokines had a high overall variability in initial start time, resulting in high standard deviations of calculated elevation starting points. However, we discovered that this variability was consistent through each cytokine elevation in the cascade. For example, if IL-10 for a given rat elevated 10 minutes earlier than our noted average, then all other cytokines would also elevate ~10 minutes earlier than our presented averages.

#### IP to IV comparisons

To compare administration methods, we utilized previously unreported cytokine data obtained from our prior research, in which we performed IV injections of LPS with an identical experimental time sequence and LPS concentration [29]. For that study, however, we extended blood collections only to 150 minutes post-LPS injection; therefore, the methods used here could be applied only to IL-10 and TNF-α. We did, however, note general elevations in GM-CSF, IL-6, and IL-22 in the chronic data. Starting point times for both IV and IP data are presented in Table 2.

**Table 2:** Comparison of cytokine elevation starting points using IP vs IV injection methods.

	Starting Point Based on 5% of Peak ± StDev	
Cytokine	Min After IP LPS Challenge (n = 44)	Min After IV LPS Challenge (n = 40)
IL-10	49.2 ± 18.6	48.1 ± 14.6
TNF-α	72.2 ± 22.9	30.3 ±7.9
GM-CSF	116.6 ± 28.4	60–90 min (n = 8)*^#^
IL-17F	125.2 ± 37.5 (n = 29)^#^	No elevations detected*
IL-6	129.6 ± 24.3 (n = 41)^#^	60–90 min (n = 40)*
IL-22	182.6 ± 33.3 (n = 26)^#^	90–120 min (n = 14)*^#^
IFN-γ	186.6 ± 25.2	No elevations detected*

*Owing to limited time points collected in IV LPS animals (0–150 min post LPS injection), specific starting points could not be properly calculated for all cytokines using our methods. Instead, a range of times in which elevations were detected, if detected, over our noise thresholds in the reduced test period is given.

^#^Had reduced number of samples showing calculable cytokine elevations.

We found there to be significant differences between temporal cytokine responses to LPS when administered IP versus IV. In general, plasma cytokine levels elevated much earlier and had significantly smaller starting point deviations in IV compared to IP. TNF-α levels showed elevations 42 minutes earlier when LPS was injected IV and showed a standard deviation almost three times smaller compared to IP administration. This disparity related to IP administration was expected and emphasizes the need for better analysis method such as those we used in this study. We noted similar earlier elevations of IL-6, GM-CSF, and IL-22 with IV-injected LPS. The times of elevations and delays are consistent with those noted in previous literature [8, 10].

Interestingly, IL-10 was a glaring exception to the standard delays noted in other cytokine elevations. Instead, we found that IL-10 had very similar elevation starting point times and standard deviations for both IV and IP LPS injection methods. IL-10 levels began elevating at around 50 minutes post LPS injection in both methods, and although IP LPS injections produced a larger deviation in elevation start time, it was only 27% larger. We found it compelling to note that this changed the order of cytokine cascades from IL-10 elevating ~23 minutes before TNF-α when using IP LPS injections, to IL-10 elevating ~18 minutes after TNF-α when injecting LPS IV. This signals a distinct physiological mechanism of the body to produce IL-10 in response to LPS, separate from that of TNF-α and other cytokines.

### Subdiaphragmatic stimulation effects

The following sections outline stimulation effects of subdiaphragmatic branch subgroups on the seven cytokine cascades that showed elevations in our study. IL-10 is primarily an anti-inflammatory cytokine; thus an increase in systemic levels is desirable. TNF-α and IL-6 are the most commonly analyzed pro-inflammatory cytokines in VNS inflammation studies and therefore it is beneficial to attenuate their levels in response to an LPS challenge. IFN-γ, GM-CSF, IL-17F, and IL-22 are all cytokines with various connections to the body’s inflammatory reflex.

We aligned data from each animal so that time “zero” corresponded to its 5% starting point and then plotted the cytokine cascades for the duration that included samples from all animals. We then averaged the effective areas under the curves of samples in each subset for a quantitative measure of total cytokine concentration, used for statistical analysis. Overall branch stimulation results compared to the SubD Sham subgroup are presented in Table 3. More detailed quantitative analyses can be found in the following sub-section paragraphs for IL-10, IL-6, and IFN-γ, and in the supplemental data supplied for TNF-α, IL-6, GM-CSF, IL-17F, and IL-22 (S2 and S3 Appendices).

**Table 3:** Cytokine effects of subdiaphragmatic branch stimulation vs SubD sham subgroup.

		IL-10	TNF-α	IL-6	IFN-γ	GM-CSF	IL-17F	IL-22
Accessory Celiac Branch	Intact	↑*	*♦*	*↓*	↑*	*↑*	*↑*	*♦*
	Vagotomy	*↑*	*♦*	*♦*	*♦*	*♦*	*↓*	*♦*
Anterior Gastric Branch	Intact	↑*	*♦*	*↑*^**#**^	↑*	*↑*	*↑*	*↑*
	Vagotomy	*↑*	*♦*	*♦*	*♦*	*♦*	*♦*	*♦*
Hepatic Branch	Intact	↑**	*♦*	*↓*	↑**	*♦*	*↑*	*↓*
	Vagotomy	↑**	*↑*	*↑*	*↑*^**#**^	*↑*	*↑*	*↑*

Blue cells indicate increases (↑), red cells indicate decreases (↓), and yellow cells indicate no noticeable change (♦) compared to SubD Sham subgroup levels. Colors are used for simplified interpretation and are not meant to signify changes as desirable or not.

Statistically significant changes in cytokine levels compared to sham group are identified.

#p < 0.1;

*p < 0.05;

**p < 0.01.

There were a few noteworthy tendencies within our overall data. First, IL-10 levels were raised by all subdiaphragmatic stimulation subgroups over that of the sham subgroup, while TNF-α remained virtually unchanged. Second, stimulation produced more selective modulatory effects when the nerve branch was left intact versus receiving an efferent vagotomy.

#### IL-10 profiles

**Fig 6:**
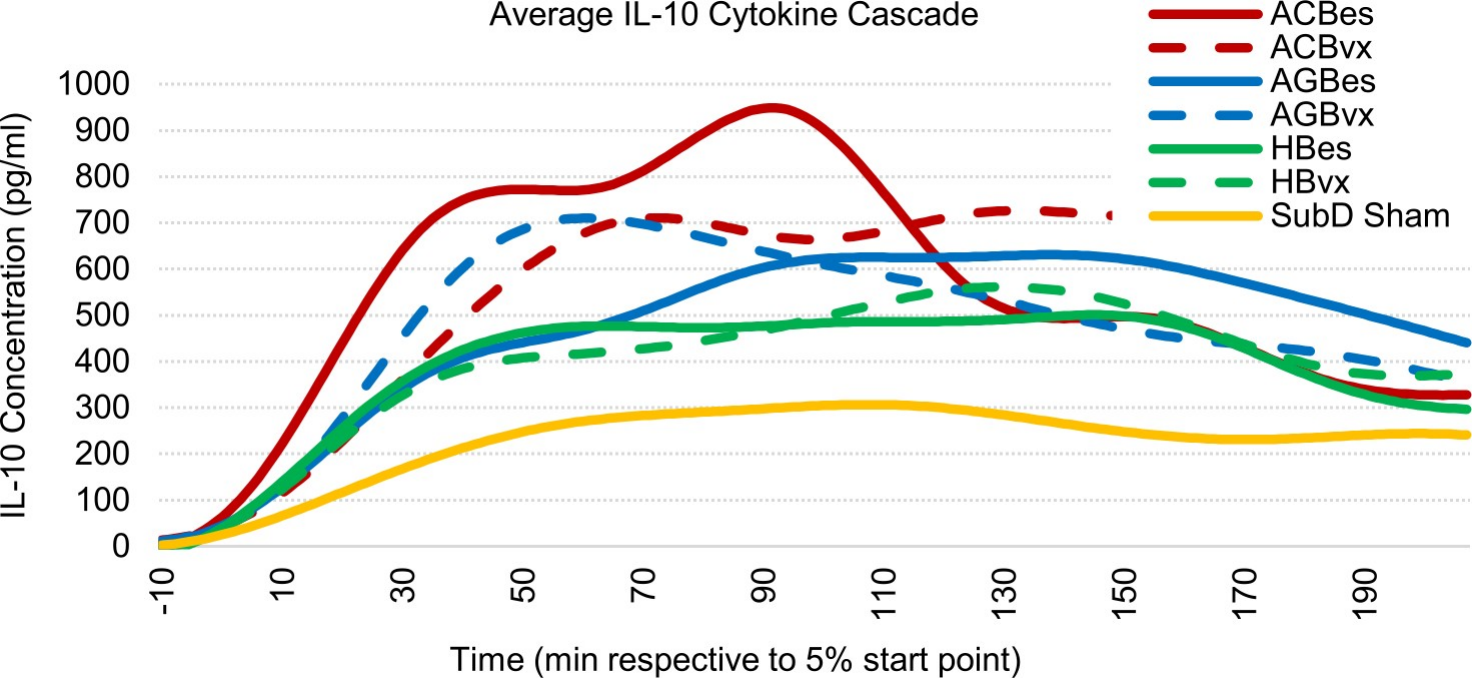
Averaged IL-10 cascade responses to IP injection of LPS (5 mg/kg). All samples were curve fitted and aligned at the time they reached 5% of peak concentration, represented as time “zero.” Time axis extends to the last time point at which all samples were still recorded.

**Fig 7:**
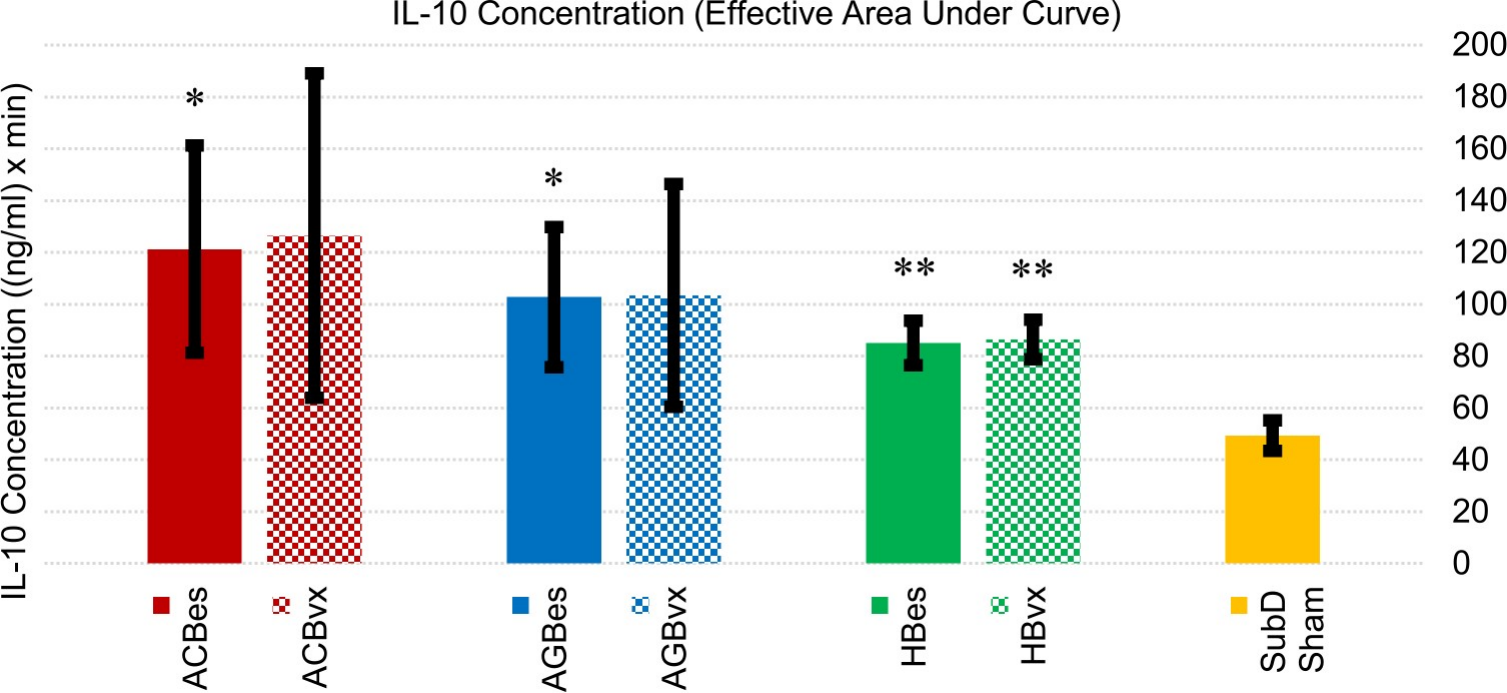
Cumulative IL-10 concentration effects of subgroup responses to IP injection of LPS (5 mg/kg). Values represent effective areas under the curve for each subgroup shown in Fig 6. Error bars, s.e.m. *p < 0.05 compared to SubD Sham group. **p < 0.01 compared to SubD Sham group.

We found IL-10 concentrations to be significantly affected by subdiaphragmatic stimulation of all three branches (Figs 6 and 7). Stimulation of the accessory celiac branch yielded highly variable IL-10 concentration responses that averaged 146% (ACBes) and 157% (ACBvx) higher than sham controls. Concentrations on the ACBes subset showed a high initial increase in IL-10 levels that dropped at the 90-minute point. However, those with efferent vagotomies showed sustained elevations of IL-10 throughout the test period. Stimulation of the anterior gastric branch also showed highly variable but clear elevations in IL-10 levels, with both subgroups averaging ~110% increases over sham control levels. The AGBvx subset showed a quicker average uptake with a decrease after ~60 minutes, while the AGBes subset had a much slower but extended uptake, not reaching a max level till after 100 minutes. Hepatic branch stimulation displayed the most consistent results, revealing statistically significant IL-10 elevations of 73% (HBes) and 75% (HBvx) over that of sham controls.

The consistency in overall response between vagotomized and non-vagotomized subsets of all three branches indicated the upturn in IL-10 response to be primarily a vagal afferent effect.

#### IL-6 profiles

**Fig 8:**
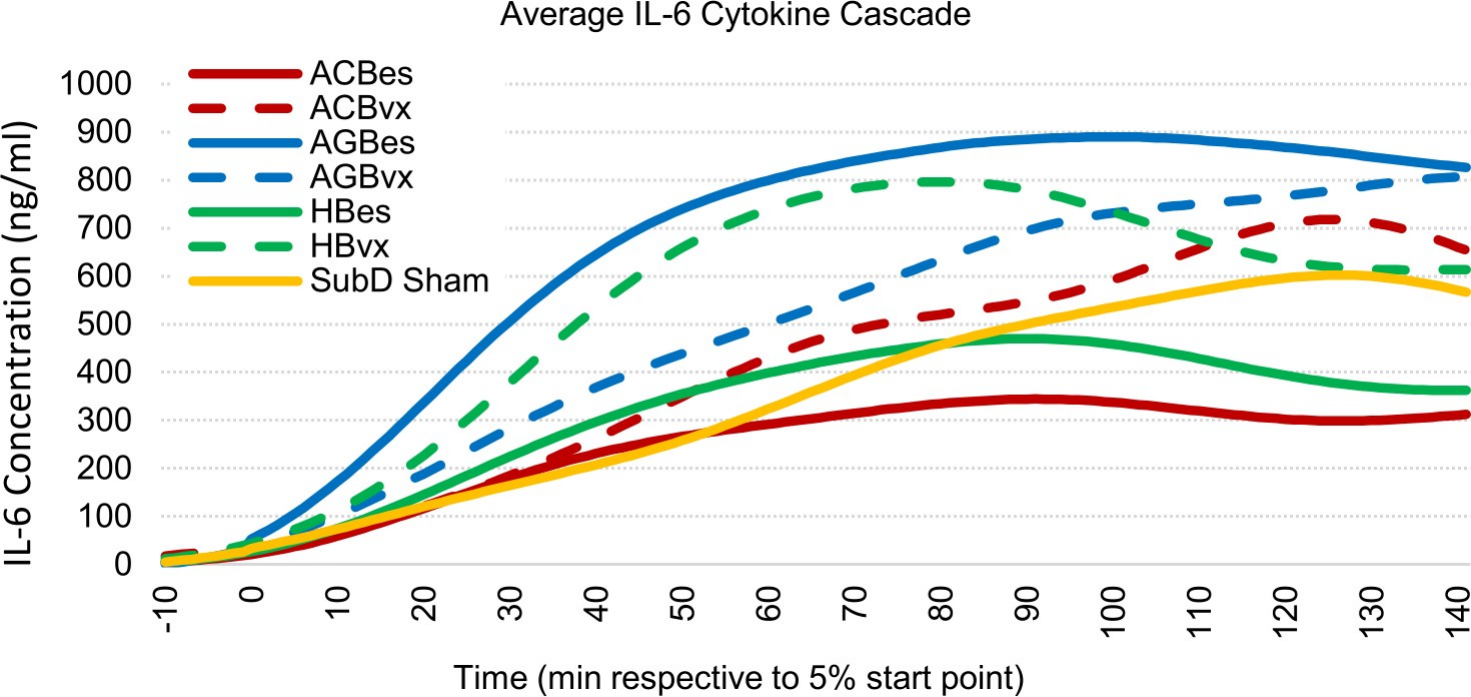
Averaged IL-6 cascade responses to IP injection of LPS (5 mg/kg). All samples were curve fitted and aligned at the time they reached 5% of peak concentration, represented as time “zero.” Time axis extends to the last time point at which all samples were still recorded.

**Fig 9:**
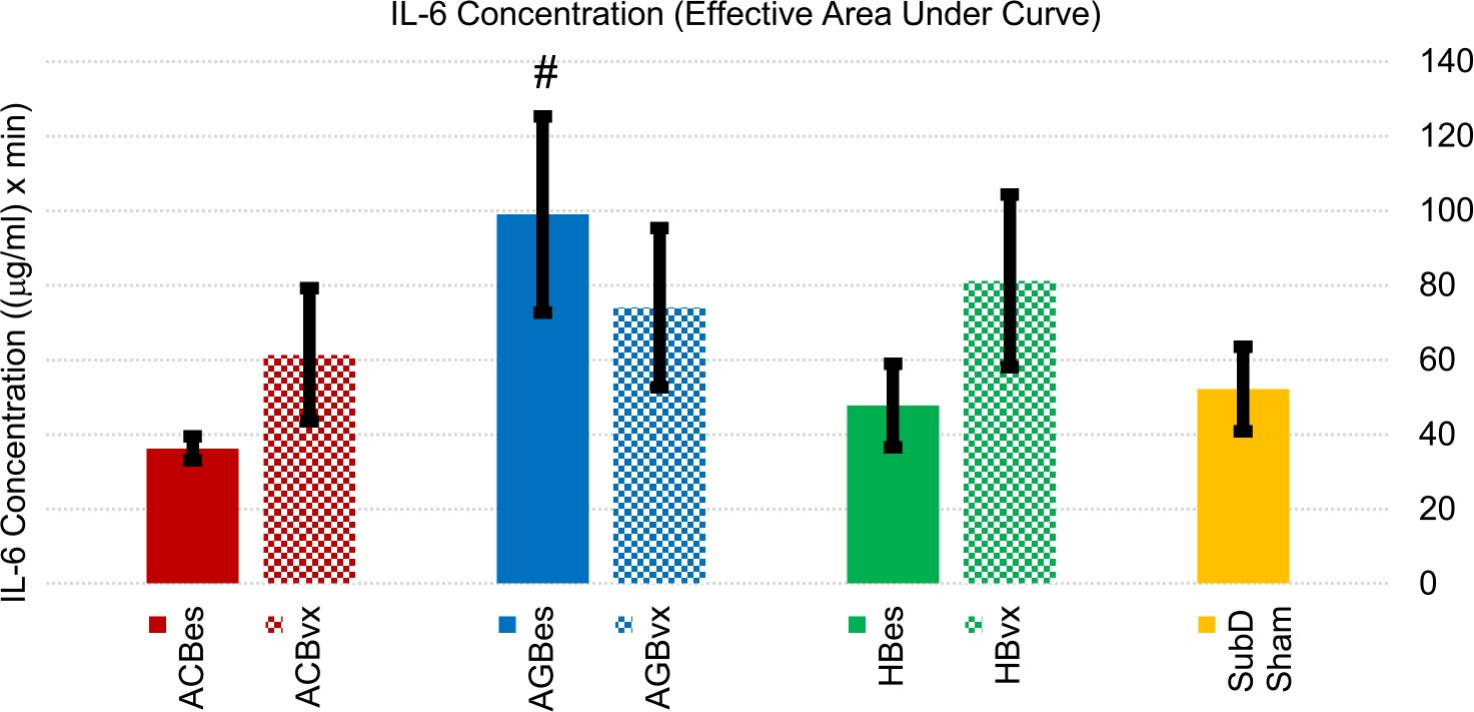
Cumulative IL-6 concentration effects of subgroup responses to IP injection of LPS (5 mg/kg). Values represent effective areas under the curve for each subgroup shown in Fig 8. Error bars, s.e.m. ^#^ p < 0.1 compared to SubD Sham group.

IL-6 effects can be seen in Figs 8 and 9. Intact anterior gastric branch stimulation produced statistically significant increases in IL-6 levels of 90% over sham controls. Of note is that multiple AGBes samples saturated the upper IL-6 limits of our flow cytometry methods, even when diluted. Therefore, the levels in that subgroup were likely even higher than reported here.

When an efferent vagotomy was performed, effects of stimulation on IL-6 changed. The increases seen from intact gastric branch stimulation were reduced, while the attenuation effects of the celiac and hepatic branch stimulation were reversed. The ACBvx subset resulted in increased IL-6 levels of ~18%, compared to a significant reduction (~30%) of IL-6 produced with the stimulation of the intact branch.

These results indicated an anti-inflammatory effect related to stimulation of the accessory celiac, and possibly, hepatic branches in which an intact efferent fiber route is present. A similar, but pro-inflammatory efferent effect was expressed by anterior gastric branch stimulation.

#### IFN-γ profiles

**Fig 10:**
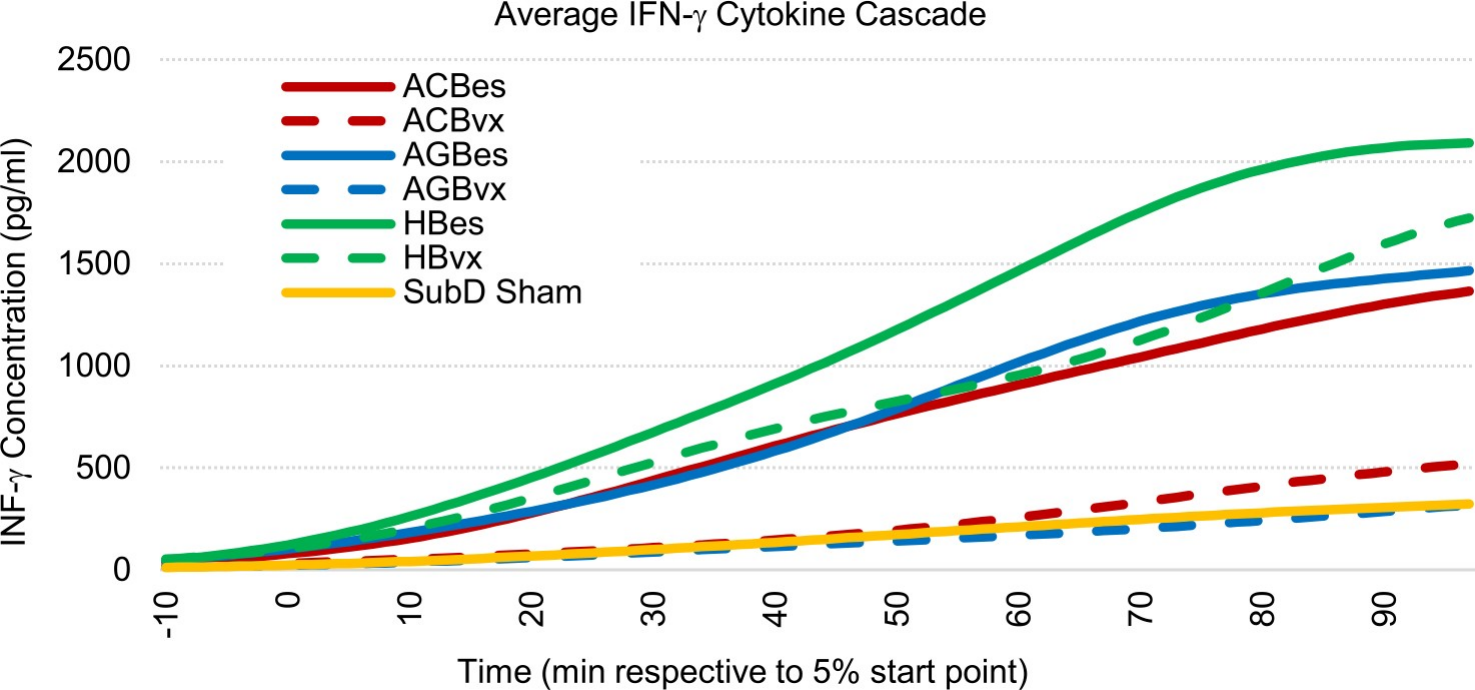
Averaged IFN-γ cascade responses to IP injection of LPS (5 mg/kg). All samples were curve fitted and aligned at the time they reached 5% of peak concentration, represented as time “zero.” Time axis extends to the last time point at which all samples were still recorded.

**Fig 11:**
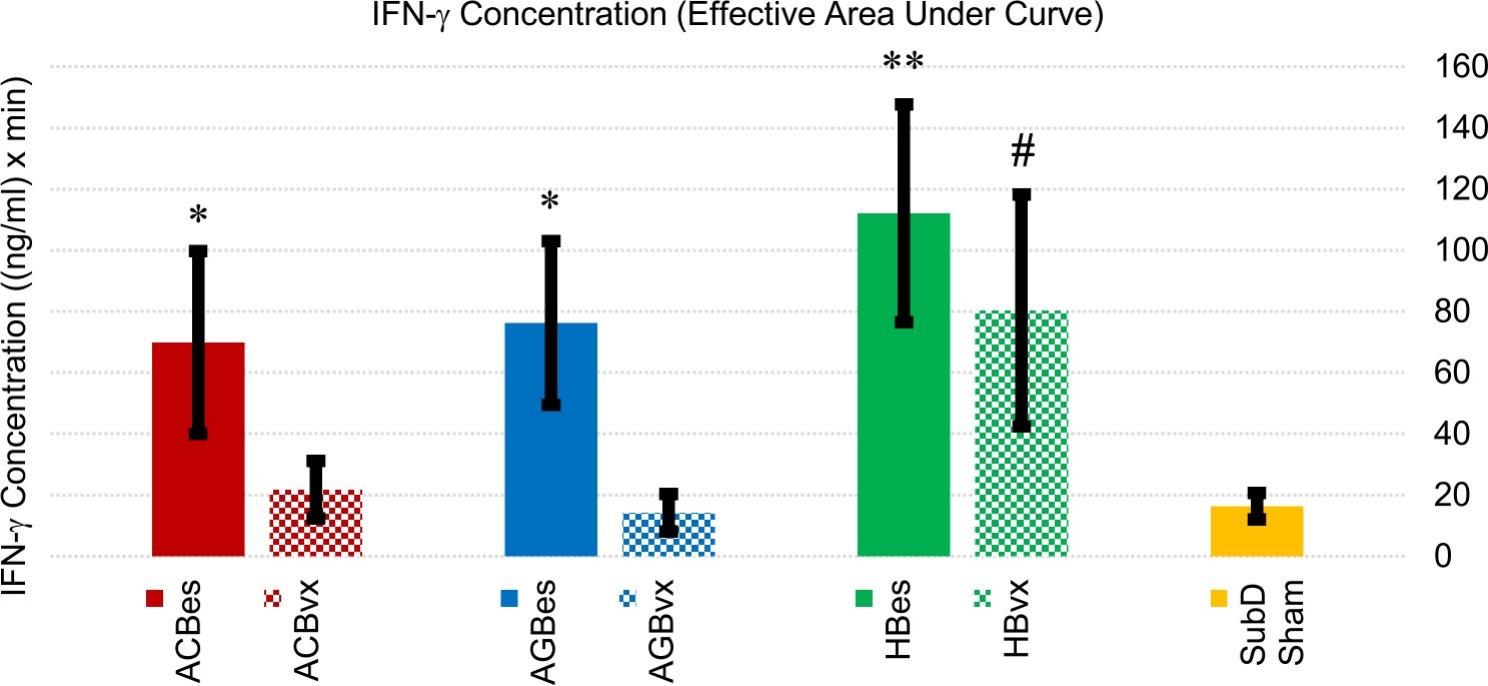
Cumulative IFN-γ concentration effects of subgroup responses to IP injection of LPS (5 mg/kg). Values represent effective areas under the curve for each subgroup shown in Fig 10. Error bars, s.e.m. ^#^ p < 0.1 compared to SubD Sham group. *p < 0.05 compared to SubD Sham group. **p < 0.01 compared to SubD Sham group.

We observed very distinct roles of several stimulation subgroups in the proliferation of IFN-γ levels in response to IP LPS challenge. IFN-γ is a less commonly analyzed pro-inflammatory cytokine that is linked to exacerbation of numerous disorders such as rheumatoid arthritis and multiple sclerosis [35]. It has also been reported that IFN-γ increases the lethality of LPS and TNF-α in mice, and correlates to increased levels of IL-6 when combined with TNF-α [36]. Owing to a lack of literature outlining VNS effects on IFN-γ levels, we believed it pertinent to emphasize our data for this cytokine. Effects are illustrated in Figs 10 and 11.

Stimulation of all three intact subdiaphragmatic branches caused escalations of IFN-γ responses to the LPS over that of sham controls. The ACBes subset showed a 327% increase, while the AGBes subset showed a 366% increase, and the HBes had the highest increase at 585% over the average level of sham controls. Meanwhile, we found that efferent vagotomies of the accessory celiac and gastric nerve branches prevented this increase. Of the vagotomy stimulation subgroups, only the hepatic afferent stimulation, by itself, significantly boosted systemic IFN-γ levels.

Our data point towards IFN-γ regulation being a predominantly efferent fiber effect, with hepatic afferents also playing a possible role.

#### Cervical electrical stimulation

Electrical stimulation of the left cervical vagus nerve was applied to contrast with subdiaphragmatic stimulation results. We did not, however, note any significant correlations between these cervical subgroups and therefore have not presented them in the main text. Instead, they are presented in the S4 Appendix with a short analysis.

## Discussion

### Temporal IP LPS–induced cytokine cascade

Through this study, we determined the temporal cascade of cytokines in response to a 5-mg/kg IP injection of LPS. To our knowledge, we are the first group to utilize our combination of consistent and frequent time points, an extended test period, and a highly diverse cytokine analysis.

For IP LPS, IL-10 is the first cytokine to rise above its baseline level, followed by TNF-α, GM-CSF, IL-17F, IL-6, IL-22 and finally IFN-γ. TNF-α has been characterized as one of the first cytokines to rise above its baseline level in several other studies [37–39] and although not believed to be the sole initiator of other cytokine responses, it has been shown to affect the concentration and duration of other cytokines [39]. The production of IL-10 is likely independent of the cascade initiated by TNF-α [38, 39], which could explain its early appearance as well as its similar timing in IP and IV LPS administrations. Additionally, it is notable that the timing of the cytokine cascade is dependent on LPS concentration administered [10], but was unaffected by stimulation in our study. Knowing the cascade of cytokines could help to target the attenuation of specific cytokines during *in-vivo* studies. For example, to effectively attenuate IFN-γ, electrical stimulation could be applied later compared to stimulation for attenuation of TNF-α.

We also analyzed inter-cytokine relationships. We found an inverse correlation between TNF-α and IL-10 levels. When IL-10 had a large spike early in the cytokine cascade, TNF-α levels were notably lower. This inverse relationship is consistent with previously reported studies [25, 40].

When comparing IP to IV LPS administration, we found much greater variability with IP administration. This was expected and has been previously reported [17, 18]. Because of this variability, it was important that we collected blood at multiple time points throughout a cytokine response, rather than at one time point, to understand the full curve for an individual animal and adjust the data accordingly. Our curve fitting and temporal shifting methods can be used by other researchers to account for variable IP absorption. Studies that evaluate cytokine concentrations at only one time point may continue to use IV LPS to reduce the variability in their data; however, we have shown evidence that the biological mechanisms are different between the two administration methods.

Typically, infections are not immediately present in the bloodstream and must be identified in the intraperitoneal space first [41]. This makes IP administration a more clinically and physiologically relevant animal model. Perhaps the most difficult aspect of utilizing an IP injection of LPS is timing of treatment. With a large variability of uptake time, it becomes challenging to match a stimulation of the nerve to the exact start time of uptake. This should not dissuade researchers, however, as there is little evidence to suggest that exact timing of a therapy with relation to uptake time has a significant impact on the effectiveness of treatment. In previous literature, various stimulation techniques, timing, and durations (even continuous stimulation in some studies) were used to effectively modulate inflammatory responses [1, 11, 16, 42, 43]. We surmise that treatment timing with relation to injection time is more important than the relation to when uptake starts. Additionally, this variability will exist clinically and serves to better model the variable state of the body when the treatment is delivered to a patient. We encourage the use of the more physiologically relevant model with thought to future clinical relevance rather than the less variable model.

There is a myriad of experimental facets that could be analyzed in an inflammatory study, such as treatment type, duration, and frequency, endotoxin type, injection method and location, as well as tissue or serum sample type and location. All of these have merit and could significantly influence the effectiveness of said treatment; however, we attempted to look at the most pertinent physiology and concentrate on those aspects and corresponding methods. Although our IV data (previously unreported) came from a chronic cuffing study in which the rats had no anesthesia during LPS challenge [29], we believe the variability between setups contributed minimally to the effects on cytokine response timing. Our data and analyses are supported by Kakizaki et al. [8], whose IV versus IP comparison showed similar delays in TNF-α and IL-6 responses, as well as more defined average peaks utilizing IV versus IP, which indicated a higher variability in the timing of IP responses.

### Inflammation regulation via subdiaphragmatic stimulation

Based on our preliminary results, the most suitable nerve branch and fibers to stimulate can be deciphered based on the need to increase or decrease each of the studied cytokine responses. However, the results of stimulation vary for each cytokine. For example, to increase IL-10 levels, the intact HB can be stimulated. This will also cause, to varying degrees, a decrease in IL-6 and IL-22, an increase in IFN-γ and IL-17F, and no change in TNF-α or GM-CSF levels. Combining stimulation and/or vagotomies of the three branches may yield desired results. This could allow for the individualization of therapy based on the required cytokine regulation.

HB stimulation and ACB stimulation had the largest overall selectivity on cytokine production. While the ACB caused changes, supporting claims made in previous work upon which our theory was founded [27], the HB induced more pronounced responses in some cases. This could be due to the larger amount of sensory fibers present in the HB (73%) compared to the ACB (69%). The AGB has only 48% sensory fibers and showed the least selectivity in modulatory effects, encouraging the idea that there is a correlation between increased sensory fiber activation and immunomodulation [44]. These interrelations suggest that there is not a singular subdiaphragmatic branch that will induce a change in all cytokine levels and thereby, similar effects can be achieved by stimulating multiple combinations of nerve branches and fibers.

The results presented support the hypothesis that while the connection of the celiac nerve branches to the spleen has been shown to be a major contributor to inflammatory cytokine reduction in sepsis and other disease models, the other subdiaphragmatic branches may also play distinct and advantageous roles. Indeed, alternate paths to inflammatory regulation involving afferent signaling and non-splenic routes have been shown in recent years [25, 45].

Afferent fibers comprise about 90% of vagal gut innervation, and a previous study by Bratton et al. implied that the connection between the vagus and spleen was indirect [13, 46], supporting our results indicating that afferent fibers can have a large influence on some cytokine levels, such as IL-10. Mapping has shown that there is a difference in termination patterns between gastric, celiac, and hepatic afferent axons in the medulla. Two major distribution patterns emerge: one due to AGB terminals and the other due to ACB and HB terminals [47]. This mapping, in conjunction with our findings, suggests that the region and density at which subdiaphragmatic afferent axon terminals occur in the medulla influence the body’s inflammatory response. Efferent nerve fibers could be activated owing to an indirect pathway or to the more commonly studied direct electrical stimulation. If fired indirectly, the various areas that the efferent fibers originate from, and the connection to those areas with respect to corresponding afferent signaling, would play a role. For example, afferent hepatic activation would lead directly to activation of hepatic efferents in the dorsal motor nucleus, and the areas that these efferent fibers innervate, versus that of the other branches. Further research is needed to confirm the efferent pathways of the ACB and HB, allowing for a deeper understanding of the role that these branches play.

Our testing can be compared to recent efforts by Komegae et al. [25], who did non-branch specific subdiaphragmatic stimulations with IV LPS administration. Most notably, we found that electric subdiaphragmatic stimulation universally increased systemic IL-10 levels over that of mere physical manipulation (SubD Sham subgroup in our study) of the subdiaphragmatic vagus. Alternately, we saw no change in TNF-α levels between those subgroups. The Komegae group, however, found matching average cytokine levels between stimulation and manipulation subgroups of both IL-10 and TNF-α. Once again, the most likely explanation for the differences in IL-10 regulation when no stimulation was applied is that the administration method had an important impact on how the body produced and modulated the cytokines, and in the effectiveness of VNS. By using our IP LPS administration methods, the cytokine response in plasma was more selectively regulated by electrical stimulation.

It is also plausible that our 100-μA pulses produced suboptimal stimulation and only activated A-type and B-type nerve fibers in those nerve branches. It is possible that a larger stimulation profile such as that used by Komegae et al. [25] or Stakenborg et al. [26] would have produced an altered attenuation, such as an increased regulation of TNF-α, by effectively stimulating additional fiber groups. The scope of this study did not allow for multiple stimulation parameters to be tested, but future research should investigate a larger variety of parameters. Additional discussion on negative results can be found in S2 and S4 Appendices.

Future research should also explore the option of stimulating individual posterior vagus nerve branches. Our study analyzed the anterior vagus nerve branches, based on cervical studies that use the left cervical vagus for anti-inflammatory VNS. The left cervical is normally chosen over the right owing to its lesser efferent effects on vital cardiological functions. However, since these functions are bypassed in the abdominal cavity it is viable that the posterior vagus nerve branches could also be utilized for distinct, and possibly superior, anti-inflammatory purposes. This is supported by Stakenborg et al. [26].

Our preliminary results demonstrate potential options for future animal and clinical research. Selective cervical efferent stimulation used in previous studies has often relied on an afferent vagotomy to yield effective inflammatory modulation [11–14]. This is a challenge to implement clinically, as surgical vagotomy causes vitamin deficiencies [48] and virtual vagotomy has not been proven in a chronic animal model or clinically to the best of our knowledge. In addition, these solutions do not account for the vagus nerve’s innervation of a vast number of organs, causing potential inherent side effects if stimulated at the cervical level. Stimulation of the anterior subdiaphragmatic branches of the left vagus nerve, and presumably, the posterior branches of the right vagus nerve, more selectively target the organs involved in cytokine production, and thus could reduce side effects compared to cervical VNS.

Chronic cuffing of the cervical vagus nerve has caused efferent fiber damage in rats, affecting the ability for cervical VNS to attenuate the inflammatory response [29]. Our results support this claim by showing higher modulatory effects when stimulating intact nerve branches over those with efferent vagotomies. However, it is possible that since certain modulatory effects of the subdiaphragmatic branches that we observed remained after efferent vagotomy, that even if the efferent fibers were damaged some anti-inflammatory effects might still be seen. The consequences of subdiaphragmatic vagotomy should be investigated to see if it is a clinically viable option to aid in selectivity of therapy. Alternative and less invasive methods could also be used to stimulate the subdiaphragmatic branches, such as focused ultrasound, which was recently used to modulate TNF-α by activating the left cervical vagus nerve [30].

## Conclusion

We have outlined new methods for interpreting cytokine production and addressing the variability in response to IP LPS, which we argue is a more suitable animal model for inflammatory diseases than IV injections. Using our methods, we have characterized the IP LPS–induced cytokine cascade and found it to follow a consistent temporal pattern with a highly variable starting time. IP and IV administrations of LPS induce different timing in the cytokine cascade for all cytokines observed except IL-10, which seems to be produced and modulated independently of other cytokines.

We also presented an analysis of the effects of individual subdiaphragmatic branch stimulation on cytokine response to IP LPS. Intact hepatic and accessory celiac branch stimulation produced the greatest modulatory effects on cytokine production following the administration of IP LPS. With the possibility of combining stimulation of various subdiaphragmatic branches and more selectively stimulating structures involved in the inflammatory reflex, use of the subdiaphragmatic branches of the left vagus nerve shows promise in the regulation of cytokines as well as further defining the inflammatory reflex. Further studies should be performed to characterize the interactions between the AGB, ACB, and HB of the vagus nerve.

## Supporting information

S1 Fig Photo of stimulation cuffs used in this study. (DOCX)

S1 Table Comparison of curves fitted with 30-minute time intervals vs with 10- or 15-minute time intervals. (DOCX)

S1 Appendix Analysis of outliers. (DOCX)

S2 Appendix TNF-α profiles. (DOCX)

S3 Appendix Remaining cytokine profiles. (DOCX)

S4 Appendix Analysis of cervical subsets. (DOCX)

## References

[1] Koopman FA, Chavan SS, Miljko S, Grazio S, Sokolovic S, Schuurman PR, et al Vagus nerve stimulation inhibits cytokine production and attenuates disease severity in rheumatoid arthritis. Proceedings of the National Academy of Sciences of the United States of America. 2016;113(29):8284–9. 10.1073/pnas.1605635113 WOS:000380224500079. 27382171 PMC4961187

[2] Bonaz B. Chronic vagus nerve stimulation in Crohn's disease: a 6- month follow- up pilot study. Neurogastroenterology And Motility: The Official Journal Of The European Gastrointestinal Motility Society. 2016;28(6):948 10.1111/nmo.12792 26920654

[3] Grimonprez A, Raedt R, Baeken C, Boon P, Vonck K. The antidepressant mechanism of action of vagus nerve stimulation: Evidence from preclinical studies. Neurosci Biobehav Rev. 2015;56:26–34. 10.1016/j.neubiorev.2015.06.019 . 26116875

[4] Groves DA, Brown VJ. Vagal nerve stimulation: a review of its applications and potential mechanisms that mediate its clinical effects. Neuroscience & Biobehavioral Reviews. 2005;29(3):493–500. 15820552 10.1016/j.neubiorev.2005.01.004

[5] Panescu D. Vagus nerve stimulation for the treatment of depression. Engineering in Medicine and Biology Magazine, IEEE. 2005;24(6):68–72. 10.1109/MEMB.2005.1549737 16382808

[6] Asari Y, Majima M, Sugimoto K, Katori M, Ohwada T. Release site of TNF alpha after intravenous and intraperitoneal injection of LPS from Escherichia coli in rats. Shock (Augusta, Ga). 1996;5(3):208–12. 10.1097/00024382-199603000-00007 8696985

[7] Fleshner M, Goehler L, Schwartz B, McGorry M, Martin D, Maier S, et al Thermogenic and corticosterone responses to intravenous cytokines (IL-1β and TNF-α) are attenuated by subdiaphragmatic vagotomy. Journal of Neuroimmunology. 1998;86(2):134–41. 9663558 10.1016/s0165-5728(98)00026-5

[8] Kakizaki Y, Watonebe H, Kohsaka A, Suda T. Temporal profiles of interleukin-1β, interleukin-6, and tumor necrosis factor-α in the plasma and hypothalamic paraven-tricular nucleus after intravenous or intraperitoneal administration of lipopolysaccharide in the rat. Endocrine Journal. 1999;46(4):487–96. 10580740 10.1507/endocrj.46.487

[9] Steven S, Dib M, Roohani S, Kashani F, Münzel T, Daiber A. Time response of oxidative/nitrosative stress and inflammation in LPS-induced endotoxaemia—A comparative study of mice and rats. International Journal of Molecular Sciences. 2017;18(10):2176. 10.3390/ijms18102176 PMC5666857 29057830

[10] Givalois L, Dornand J, Mekaouche M, Solier M, Bristow A, Ixart G, et al Temporal cascade of plasma level surges in ACTH, corticosterone, and cytokines in endotoxin-challenged rats. American Journal of Physiology-Regulatory, Integrative and Comparative Physiology. 1994;267(1):R164–R70. 10.1152/ajpregu.1994.267.1.R164 8048620

[11] Borovikova LV, Ivanova S, Zhang MH, Yang H, Botchkina GI, Watkins LR, et al Vagus nerve stimulation attenuates the systemic inflammatory response to endotoxin. Nature. 2000;405(6785):458–62. WOS:000087212000049. 10.1038/35013070 10839541

[12] Huston JM, Ochani M, Rosas-Ballina M, Liao H, Ochani K, Pavlov VA, et al Splenectomy inactivates the cholinergic antiinflammatory pathway during lethal endotoxemia and polymicrobial sepsis. J Exp Med. 2006;203(7):1623–8. 10.1084/jem.20052362 16785311 PMC2118357

[13] Bratton BO, Martelli D, McKinley MJ, Trevaks D, Anderson CR, McAllen RM. Neural regulation of inflammation: no neural connection from the vagus to splenic sympathetic neurons. Experimental Physiology. 2012;97(11):1180–5. 10.1113/expphysiol.2011.061531 22247284

[14] Patel YA, Saxena T, Bellamkonda RV, Butera RJ. Kilohertz frequency nerve block enhances anti-inflammatory effects of vagus nerve stimulation. Scientific Reports. 2017;7:39810 10.1038/srep39810 28054557 PMC5215548

[15] Wang H, Yu M, Ochani M, Amella CA, Tanovic M, Susarla S, et al Nicotinic acetylcholine receptor α7 subunit is an essential regulator of inflammation. Nature. 2003;421(6921):384 10.1038/nature01339 12508119

[16] Rosas-Ballina M, Olofsson PS, Ochani M, Valdes-Ferrer SI, Levine YA, Reardon C, et al Acetylcholine- synthesizing T cells relay neural signals in a vagus nerve circuit. Science. 2011;334(6052):98–101. 10.1126/science.1209985 21921156 PMC4548937

[17] Lenczowski M, Van Dam A-M, Poole S, Larrick J, Tilders F. Role of circulating endotoxin and interleukin-6 in the ACTH and corticosterone response to intraperitoneal LPS. American Journal of Physiology-Regulatory, Integrative and Comparative Physiology. 1997;273(6):R1870–R7. 10.1152/ajpregu.1997.273.6.R1870 9435639

[18] Lenczowski M, Schmidt E, DAM AM, Gaykema R, Tilders F. Individual variation in hypothalamus‐pituitary‐adrenal responsiveness of rats to endotoxin and interleukin‐1β. Annals of the New York Academy of Sciences. 1998;856(1):139–47. 9917874 10.1111/j.1749-6632.1998.tb08322.x

[19] Tracey K. The inflammatory reflex. Nature 2002 p. 853–9. 10.1038/nature01321 12490958

[20] Bellinger DL, Lorton D, Hamill RW, Felten SY, Felten DL. Acetylcholinesterase staining and choline acetyltransferase activity in the young adult rat spleen: Lack of evidence for cholinergic innervation. Brain Behavior and Immunity. 1993;7(3):191–204. 10.1006/brbi.1993.1021 8219410

[21] Berthoud HR, Carlson NR, Powley TL. Topography of efferent vagal innervation of the rat gastrointestinal tract. The American Journal of Physiology. 1991;260(1 Pt 2):R200. 1992820 10.1152/ajpregu.1991.260.1.R200

[22] Martelli D, McKinley MJ, McAllen RM. The cholinergic anti-inflammatory pathway: A critical review. Autonomic Neuroscience-Basic & Clinical. 2014;182:65–9. 10.1016/j.autneu.2013.12.007 WOS:000335634600006. 24411268

[23] Berthoud H-R, Neuhuber WL. Functional and chemical anatomy of the afferent vagal system. Autonomic Neuroscience. 2000;85(1):1–17. 11189015 10.1016/S1566-0702(00)00215-0

[24] Huston JM, Ochani M, Ochani K, Rosas-Ballina M, Gallowitsch-Puerta M, Yang L, et al Splenectomy protects against sepsis lethality and reduces serum HMGB1 levels. Journal of Immunology. 2008;181(5):3535–9. 10.4049/jimmunol.181.5.3535 PMC4533852 18714026

[25] Komegae EN, Farmer DGS, Brooks VL, McKinley MJ, McAllen RM, Martelli D. Vagal afferent activation suppresses systemic inflammation via the splanchnic anti-inflammatory pathway. Brain, Behavior, and Immunity. 2018. 10.1016/j.bbi.2018.06.005 PMC6319822 29883598

[26] Stakenborg N, Wolthuis A, Gomez‐Pinilla PJ, Farro G, Di Giovangiulio M, Bosmans G, et al Abdominal vagus nerve stimulation as a new therapeutic approach to prevent postoperative ileus. Neurogastroenterology & Motility. 2017;29(9):e13075. 10.1111/nmo.13075 28429863

[27] Berthoud H-R, Powley TL. Characterization of vagal innervation to the rat celiac, suprarenal and mesenteric ganglia. Journal of the Autonomic Nervous System. 1993;42(2):153–69. 10.1016/0165-1838(93)90046-W 8450174

[28] Berthoud HR, Powley TL. Interaction between parasympathetic and sympathetic nerves in prevertebral ganglia: morphological evidence for vagal efferent innervation of ganglion cells in the rat. Microscopy Research and Technique. 1996;35(1):80 10.1002/(SICI)1097-0029(19960901)35:1<80::AID-JEMT7>3.0.CO;2-W 8873061

[29] Somann JP, Albors G, Neihouser K, Lu K-H, Liu Z, Ward MP, et al Chronic cuffing of cervical vagus nerve inhibits efferent fiber integrity in rat model. Journal of Neural Engineering. 2017. 10.1088/1741-2552/aaa039 PMC7493020 29219123

[30] Wasilczuk KM, Bayer KC, Somann JP, Albors GO, Sturgis J, Lyle LT, et al Modulating the inflammatory reflex in rats using low-intensity focused ultrasound stimulation of the vagus nerve. Ultrasound in Medicine & Biology. 2019;45(2):481–9. 30396599 10.1016/j.ultrasmedbio.2018.09.005

[31] Al-Mousawi AM, Kulp GA, Branski LK, Kraft R, Mecott GA, Williams FN, et al Impact of anesthesia, analgesia and euthanasia technique on the inflammatory cytokine profile in a rodent model of severe burn injury. Shock (Augusta, Ga). 2010;34(3):261. 10.1097/shk.0b013e3181d8e2a6 PMC3827633 20803788

[32] Bioanalytical Systems I. Surgical Procedures: Femoral Cannulation (Rat). Surgery Manual. 2007;Culex ABS.

[33] Peters S, Hampsch J, Cregor M, Starrett C, Gunaratna G, Kissinger C. Culex ABS Part I: Introduction to automated blood sampling. Current Separations. 2000;18(4):140.

[34] Jarry KV. An exploratory study of how acute neuromodulation of the subdiaphragmatic branches regulates inflammation. Purdue University. Biomedical Engineering. degree granting i, editor: Ann Arbor: ProQuest Dissertations & Theses; 2017.

[35] Kraaij MD, Vereyken EJ, Leenen PJ, van den Bosch TP, Rezaee F, Betjes MG, et al Human monocytes produce interferon-gamma upon stimulation with LPS. Cytokine. 2014;67(1):7–12. 10.1016/j.cyto.2014.02.001 24680476

[36] Doherty GM, Lange JR, Langstein HN, Alexander HR, Buresh CM, Norton JA. Evidence for IFN-gamma as a mediator of the lethality of endotoxin and tumor necrosis factor-alpha. The Journal of Immunology. 1992;149(5):1666–70. 1506688

[37] Redl H, Schlag G, Bahrami S, Schade U, Ceska M, Stutz P. Plasma neutrophil-activating peptide-1/interleukin-8 and neutrophil elastase in a primate bacteremia model. Journal of Infectious Diseases. 1991;164(2):383–8. 10.1093/infdis/164.2.383 1906912

[38] Suffredini AF, Reda D, Banks SM, Tropea M, Agosti JM, Miller R. Effects of recombinant dimeric TNF receptor on human inflammatory responses following intravenous endotoxin administration. Journal of Immunology. 1995;155(10):5038–45. 7594512

[39] Gogos Charalambos A, Drosou E, Bassaris Harry P, Skoutelis A. Pro‐ versus anti‐inflammatory cytokine profile in patients with severe sepsis: A marker for prognosis and future therapeutic options. The Journal of Infectious Diseases. 2000;181(1):176–80. 10.1086/315214 10608764

[40] van der Poll T, Jansen J, Levi M, ten Cate H, ten Cate JW, van Deventer SJ. Regulation of interleukin 10 release by tumor necrosis factor in humans and chimpanzees. The Journal of Experimental Medicine. 1994;180(5):1985–8. 7964475 10.1084/jem.180.5.1985 PMC2191735

[41] Medzhitov R. Origin and physiological roles of inflammation. Nature. 2008;454(7203):428 10.1038/nature07201 18650913

[42] Meregnani J, Clarencon D, Vivier M, Peinnequin A, Mouret C, Sinniger V, et al Anti-inflammatory effect of vagus nerve stimulation in a rat model of inflammatory bowel disease. Autonomic Neuroscience. 2011;160(1–2):82–9. 10.1016/j.autneu.2010.10.007 . 21071287

[43] Chapleau MW, Rotella DL, Reho JJ, Rahmouni K, Stauss HM. Chronic vagal nerve stimulation prevents high-salt diet-induced endothelial dysfunction and aortic stiffening in stroke-prone spontaneously hypertensive rats. Am J Physiol Heart Circ Physiol. 2016;311(1):H276–85. 10.1152/ajpheart.00043.2016 27208157 PMC4967207

[44] Prechtl JC, Powley TL. The fiber composition of the abdominal vagus of the rat. Anatomy and Embryology. 1990;181(2):101–15. 10.1007/BF00198950 2327594

[45] Matteoli G, Gomez-Pinilla PJ, Nemethova A, Di Giovangiulio M, Cailotto C, van Bree SH, et al A distinct vagal anti-inflammatory pathway modulates intestinal muscularis resident macrophages independent of the spleen. Gut. 2013:gutjnl-2013-304676. 10.1136/gutjnl-2013-304676 23929694

[46] Strominger NL, Demarest RJ, Laemle LB. Noback's human nervous system: structure and function: Springer Science & Business Media; 2012.

[47] Norgren R, Smith GP. Central distribution of subdiaphragmatic vagal branches in the rat. Journal of Comparative Neurology. 1988;273(2):207–23. 10.1002/cne.902730206 3417902

[48] Streeter AM, Duraiappah B, Boyle R, O'Neill BJ, Pheils MT. Malabsorption of vitamin B12 after vagotomy. The American Journal of Surgery. 1974;128(3):340–3. 4414546 10.1016/0002-9610(74)90169-x

