# Supplementary material for: Characterization of plasma cytokine response to intraperitoneally administered LPS & subdiaphragmatic branch vagus nerve stimulation in rat model
Source: PLoS One. 2019 Mar 28;14(3):e0214317. doi: 10.1371/journal.pone.0214317 (PMC6438475; doi:10.1371/journal.pone.0214317)
Supplement: S1 Fig — (DOCX) [file pone.0214317.s001.docx]

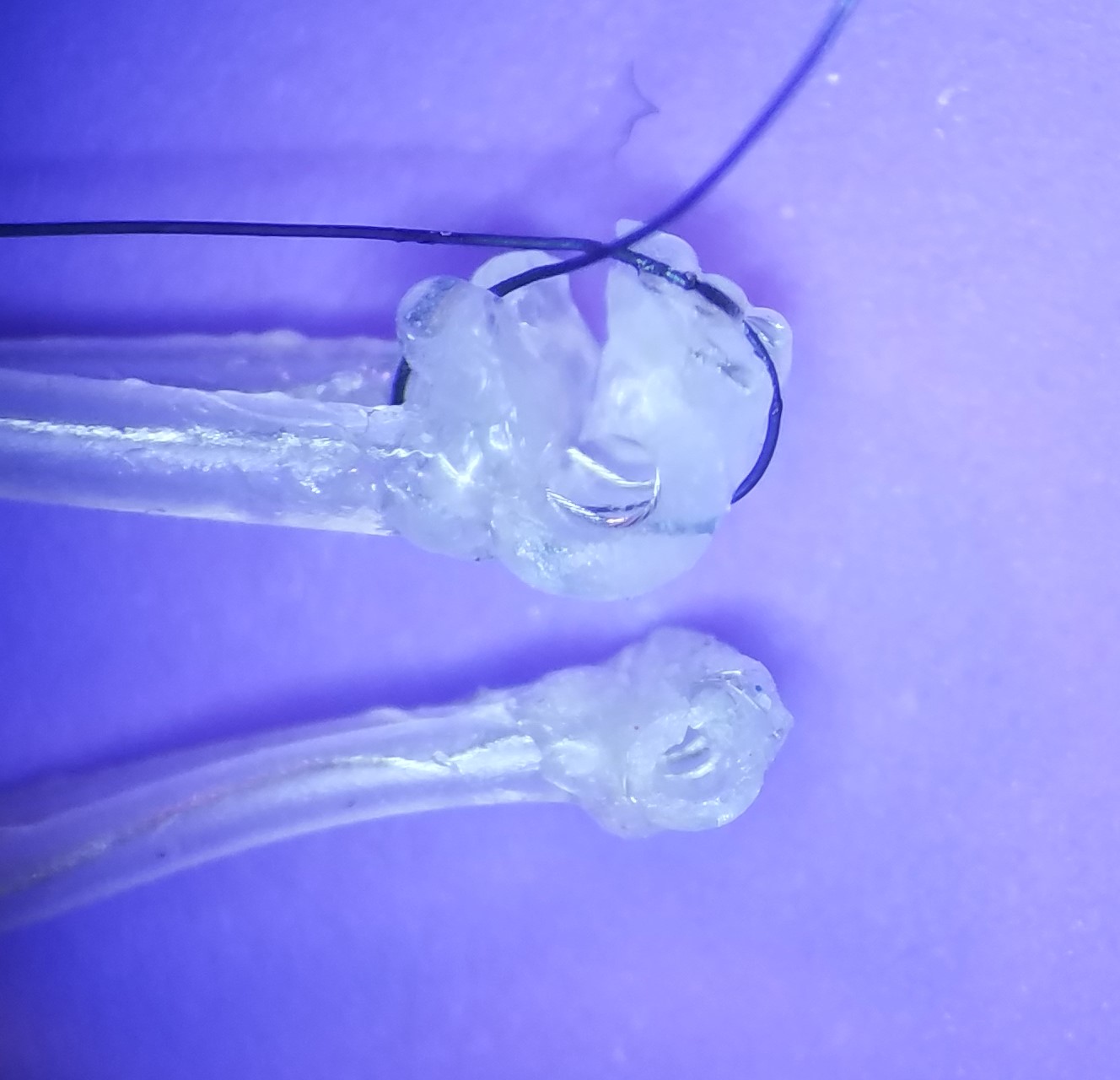


**S1 Fig**. **Photo of stimulation cuffs used in this study**. Larger cuff for cervical vagus nerve stimulation is shown on top with built-in suture to fasten it around a nerve. Smaller cuff for subdiaphragmatic nerve branch stimulation is shown on the bottom.
