# Supplementary material for: Characterization of plasma cytokine response to intraperitoneally administered LPS & subdiaphragmatic branch vagus nerve stimulation in rat model
Source: PLoS One. 2019 Mar 28;14(3):e0214317. doi: 10.1371/journal.pone.0214317 (PMC6438475; doi:10.1371/journal.pone.0214317)
Supplement: S1 Table — (DOCX) [file pone.0214317.s002.docx]

**S1 Table. Comparison of curves fitted with 30-minute time intervals vs with 10- or 15-minute time intervals.**

|  | **Average Difference of 5% of Max Peak Starting Points** | **Average Percent Difference of Area Under Curves** |
| --- | --- | --- |
| **Cytokine** | Minutes ± StDev | Percent Difference ± StDev |
| IL-10 | -4.3 ± 4.1 (n = 11) | -2.6% ± 9.0% (n = 11) |
| TNF-α | +0.6 ± 2.3 (n = 11) | +0.5% ± 5.2% (n = 11) |
| GM-CSF | -3.8 ± 4.6 (n = 6) | -2.9% ± 9.7% (n = 6) |
| IL-17F | No elevations detected* | No elevations detected* |
| IL-6 | -2.6 ± 3.4 (n = 11) | +2.0% ± 14.1% (n = 11) |
| IL-22 | +3.3 ± 7.1 (n = 3) | +2.7% ± 9.7% (n = 3) |
| IFN-γ | +0.7 ± 2.9 (n = 3) | +1.3% ± 13.1% (n = 3) |

**No validation test animals had sufficient levels of IL-17F elevation for analysis.*
