# Supplementary material for: Characterization of plasma cytokine response to intraperitoneally administered LPS & subdiaphragmatic branch vagus nerve stimulation in rat model
Source: PLoS One. 2019 Mar 28;14(3):e0214317. doi: 10.1371/journal.pone.0214317 (PMC6438475; doi:10.1371/journal.pone.0214317)
Supplement: S1 Appendix — (DOCX) [file pone.0214317.s003.docx]

**S1 Appendix. Analysis of outliers.**

**S1 Appendix Table. List of Outliers Omitted from Formal Data Analysis.**

| **Subgroup** | **Reason for Omission from Results** |
| --- | --- |
| ACBes | No elevated cytokine levels after IP LPS injection |
| ACBes | No elevated cytokine levels after IP LPS injection |
| ACBvx | No cytokine elevation in 5 of 7 cytokines, statistically low outlier in remaining cytokines |
| HBes | No elevated cytokine levels after IP LPS injection |
| AGBns | Statistically high outlier in 4 of 7 cytokines, abnormally high levels in remaining cytokines |
| CVns | Surgical anomaly, animal showed abnormally high heart rates prior to LPS administration and throughout experiment |
| CVns | Surgical anomaly, animal struggled to breath throughout experiment and had to be revived twice |

Of important note in our study is that four out of 55 rats showed little or no response in any cytokine levels after IP LPS administration. While we thought it most likely that this was a biological anomaly in these animals similar to those experienced by Lenczowski et al [1, 2], it is interesting to note that our rate of unresponsive animals was significantly less than that experienced by them. It is curious that their last data point collected was at 90 minutes post IP LPS injection, a time point in which ~20% of our animals had yet to show significant elevations of IL-6, the cytokine of interest in the Lenczowski studies. We hypothesize that had they collected samples at a later time-point, that they would have seen IL-6 elevations in a larger number of their animals. It is also interesting to note that none of our nonresponsive animals were in sham subgroups and all four were in stimulation subgroups that showed significant amounts of cytokine modulation in responsive rats (two ACBes, one ACBvx, and one HBes). Therefore, while the attenuation effect was dramatic in these four animals and we considered them outliers for statistical purposes, it is possible that the stimulation applied to these animals did indeed play some part in the absence of inflammatory response.

1. Lenczowski M, Van Dam A-M, Poole S, Larrick J, Tilders F. Role of circulating endotoxin and interleukin-6 in the ACTH and corticosterone response to intraperitoneal LPS. American Journal of Physiology-Regulatory, Integrative and Comparative Physiology. 1997;273(6):R1870-R7.

2. Lenczowski M, Schmidt E, DAM AM, Gaykema R, Tilders F. Individual variation in hypothalamus‐pituitary‐adrenal responsiveness of rats to endotoxin and interleukin‐1β. Annals of the New York Academy of Sciences. 1998;856(1):139-47.
