# Supplementary material for: Characterization of plasma cytokine response to intraperitoneally administered LPS & subdiaphragmatic branch vagus nerve stimulation in rat model
Source: PLoS One. 2019 Mar 28;14(3):e0214317. doi: 10.1371/journal.pone.0214317 (PMC6438475; doi:10.1371/journal.pone.0214317)
Supplement: S2 Appendix — (DOCX) [file pone.0214317.s004.docx]

**S2 Appendix. TNF-α profiles.**

While responses had high variability in all subsets that we sampled and did not show statistically significant trends, we have included it in our results to stay consistent with prior literature and further demonstrate our methods. Effects are illustrated in S4 Figs 1 and 2.

Stimulation of the intact nerve branches appeared to have little effect on the production of TNF-α. AGBes and HBes subgroups showed responses that were only 17% higher than sham control, while ACBes was nearly identical to sham. Stimulation of the accessory celiac and gastric nerve branches with efferent vagotomies exhibited virtually identical overall TNF-α responses, with the ACBvx subset appearing to have a slightly slower rate of increase. HBvx stimulation showed the largest effect on TNF-α levels, displaying a higher peak concentration and an overall average concentration 42% higher than sham controls.


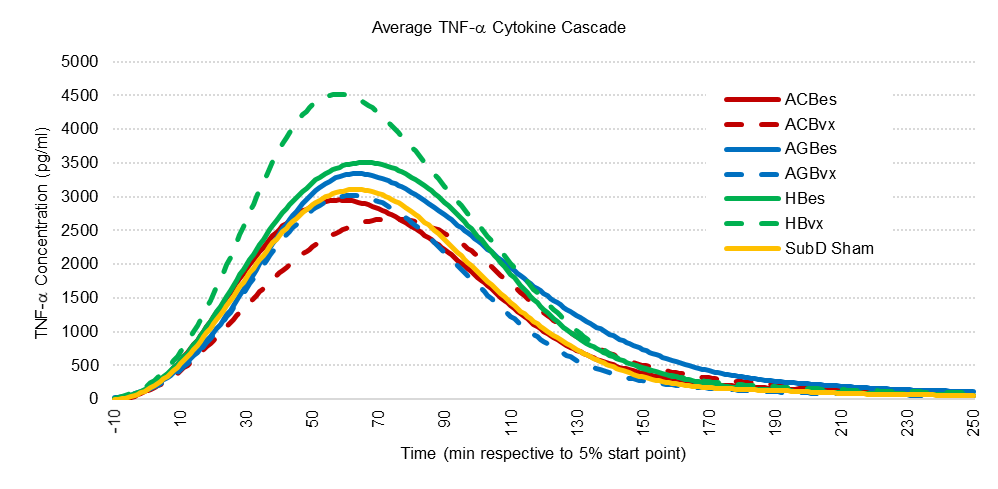
**S2 Fig 1. Averaged TNF-α cascade responses to IP injection of LPS (5 mg/kg**). All samples were curve fitted and aligned at the time they reached 5% of peak concentration, represented as time “zero.” Time axis extends to the last time point in which all samples were still recorded.


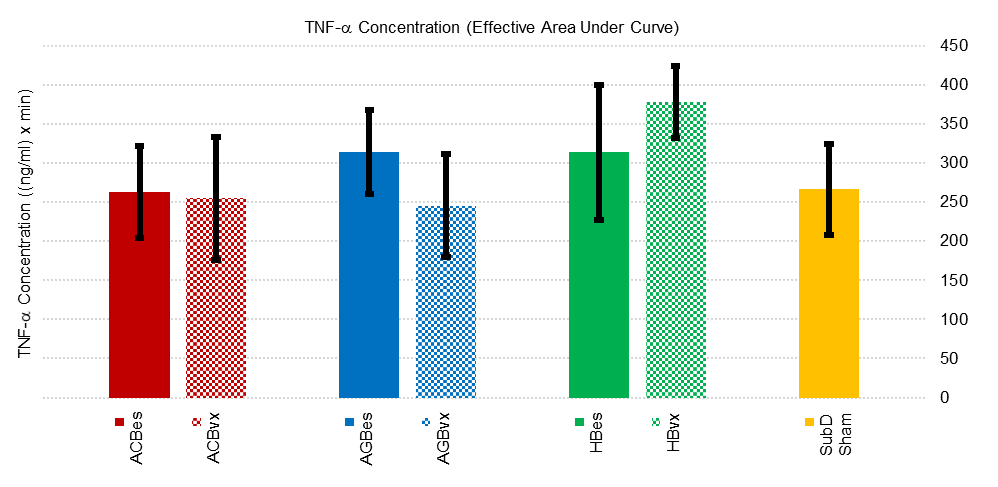
**S2 Fig 2**. **Cumulative TNF-α concentration effects of subgroup responses to IP injection of LPS (5 mg/kg).** Values represent effective areas under the curve corresponding to time points illustrated in S4 Fig 1. Error bars, s.e.m.

With respect to a lack of significant reduction of TNF-α levels, as opposed to prior research that has effectively achieved reductions, the most likely explanation is that our stimulation parameters used on the subdiaphragmatic branches were sub-optimal. The use of 100 µA pulses is significantly lower than prior studies such as Stakenborg et al. [1] who used full 1 mA pulses. It is possible that while affecting production of the cytokines discussed in the primary manuscript, that 100 µA did not fully activate the fibers required for effective attenuation of TNF-α.

There is the also the possibility that afferently attenuated effects could have offset effective cytokine attenuation. We did not perform afferent cervical vagotomy subsets for this study, but many studies that have performed cervical VNS studies on rats have relied on afferent vagotomies of the cervical vagus, rostral to the stimulation, in order to achieve significant inflammatory attenuation [2-6].

1. Stakenborg N, Wolthuis A, Gomez‐Pinilla PJ, Farro G, Di Giovangiulio M, Bosmans G, et al. Abdominal vagus nerve stimulation as a new therapeutic approach to prevent postoperative ileus. Neurogastroenterology & Motility. 2017;29(9):e13075.

2. Borovikova LV. Vagus nerve stimulation attenuates the systemic inflammatory response to endotoxin. Nature. 2000;405(6785):458-63.

3. Huston JM, Ochani M, Rosas-Ballina M, Liao H, Ochani K, Pavlov VA, et al. Splenectomy inactivates the cholinergic antiinflammatory pathway during lethal endotoxemia and polymicrobial sepsis. J Exp Med. 2006;203(7):1623-8.

4. Rosas-Ballina M, Ochani M, Parrish WR, Ochani K, Harris YT, Huston JM, et al. Splenic nerve is required for cholinergic antiinflammatory pathway control of TNF in endotoxemia. Proceedings of the National Academy of Sciences of the United States of America. 2008;105(31):11008. doi: 10.1073/pnas.0803237105.

5. Bratton BO, Martelli D, McKinley MJ, Trevaks D, Anderson CR, McAllen RM. Neural regulation of inflammation: no neural connection from the vagus to splenic sympathetic neurons. Experimental Physiology. 2012;97(11):1180-5. doi: 10.1113/expphysiol.2011.061531.

6. Patel YA, Saxena T, Bellamkonda RV, Butera RJ. Kilohertz frequency nerve block enhances anti-inflammatory effects of vagus nerve stimulation. Scientific Reports. 2017;7:39810. doi: 10.1038/srep39810.
