# Supplementary material for: Characterization of plasma cytokine response to intraperitoneally administered LPS & subdiaphragmatic branch vagus nerve stimulation in rat model
Source: PLoS One. 2019 Mar 28;14(3):e0214317. doi: 10.1371/journal.pone.0214317 (PMC6438475; doi:10.1371/journal.pone.0214317)
Supplement: S3 Appendix — (DOCX) [file pone.0214317.s005.docx]

**S3 Appendix. Remaining cytokine profiles.**

GM-CSF is typically linked to allergic inflammation, but presents with a wide range of pathologies [1], while IL-17F acts as a pro-inflammatory cytokine often with relation to lung inflammation [2] and IL-22 has been shown to play both a positive and negative role in different immune disorders [3]. Thereby, regulation of any or all three of these specific cytokines could yield new therapy options for inflammatory diseases.

Statistical quantitative analysis of elevations for these cytokines was unreasonable due to respectively low concentration levels and inconsistent rises. Instead, we did a simple fractional analysis of samples that rose above a nominal noise threshold for our flow cytometry methods that is shown in Table 3. In doing so, we found qualitative trends of note between subgroups.

Accessory celiac branch stimulation was consistently effective at modulating both GM-CSF and IL-17F, but the consistency of elevations was reduced when the branch was given an efferent vagotomy. Simulation of the intact gastric branch also showed increased upregulation of all three cytokines, while an efferent vagotomy to the branch prevented these increases. The hepatic branch showed the most effective regulatory ability. Stimulation of the intact hepatic branch (HBes subgroup) was effective at elevating IL-17F levels, but not GM-CSF or IL-22. However, when an efferent hepatic vagotomy (HBvx subgroup) was performed, stimulation showed increased frequency of elevations in all three cytokines.

**S3 Table. Fractional Analysis of Cytokine Cascades Over Given Threshold Concentrations.**

| **Cytokine** | **GM-CSF** | **IL-17F** | **IL-22** |
| --- | --- | --- | --- |
| Threshold | 80 pg/ml | 100 pg/ml | 100 pg/ml |
| **Subset** | | | |
| ACBes | 3/5 | 3/5 | 2/5 |
| ACBvx | 2/4 | 0/4 | 2/4 |
|  | | | |
| AGBes | 3/5 | 3/5 | 3/5 |
| AGBvx | 2/4 | 1/4 | 1/4 |
|  | | | |
| HBes | 1/4 | 4/4 | 0/4 |
| HBvx | 3/5 | 4/5 | 4/5 |
|  | | | |
| SubD Sham | 2/8 | 3/8 | 1/8 |

*Fractions are the number of animals that had cytokine cascade elevations above the given threshold.*

*Colors are used for simplified interpretation and are not meant to signify results as desirable or not. Blue cells are subsets that had over half its samples rise above threshold. Yellow cells are subsets that had half or less of its samples rise above threshold. Red cells are subsets in which none if its samples rose above threshold.*

1. Hamilton JA, Anderson GP. Mini ReviewGM-CSF Biology. Growth factors. 2004;22(4):225-31.

2. You Q-h, Sun G-y, Wang N, Shen J-l, Wang Y. Interleukin-17F-induced pulmonary microvascular endothelial monolayer hyperpermeability via the protein kinase C pathway. Journal of Surgical Research. 2010;162(1):110-21.

3. Pan H-F, Li X-P, Zheng SG, Ye D-Q. Emerging role of interleukin-22 in autoimmune diseases. Cytokine & growth factor reviews. 2013;24(1):51-7.
