# Supplementary material for: Characterization of plasma cytokine response to intraperitoneally administered LPS & subdiaphragmatic branch vagus nerve stimulation in rat model
Source: PLoS One. 2019 Mar 28;14(3):e0214317. doi: 10.1371/journal.pone.0214317 (PMC6438475; doi:10.1371/journal.pone.0214317)
Supplement: S4 Appendix — (DOCX) [file pone.0214317.s006.docx]

**S4 Appendix. Analysis of cervical subsets.**

Cervical VNS showed variable levels of overall cytokine modulation in response to an IP LPS challenge as can be seen in S6 Fig 1. There appeared to be varying attenuation effects of cervical stimulation on levels of TNF-α, IL-10, and IFN-γ in comparison to non-stimulated animals, while there were virtually no changes in IL-6 levels.

**
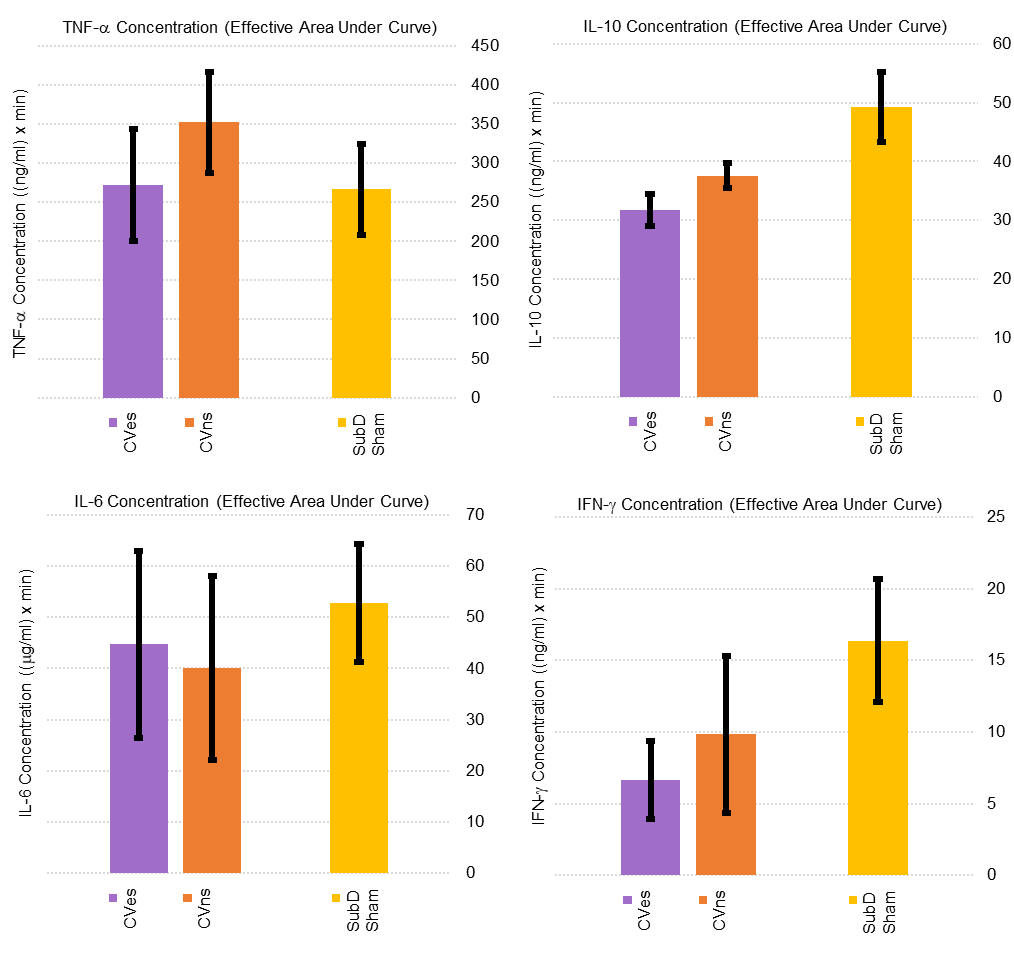
**

**S4 Fig 1**. **Cumulative cytokine concentration effects of cervical subgroup responses to IP injection of LPS (5 mg/kg).** Left Cervical Vagus Stimulation (CVes) and Sham (CVns) subgroups are represented. Subdiaphragmatic Sham (SubD Sham) group is included for comparison. All samples were curve fitted and aligned at the time they reached 5% of peak concentration, as was done with subdiaphragmatic subsets. Values given here represent effective areas under the curve for each subgroup. Error bars, s.e.m. No statistical increases or reductions in cytokine levels were observed.

With respect to a lack of statistically significant reduction of inflammatory cytokine levels, there is the possibility that afferently attenuated effects could have offset effective cytokine attenuation. We did not perform afferent cervical vagotomy subsets for this study to stay consistent with our subdiaphragmatic subgroups, but many studies that have performed cervical VNS studies on rats have relied on afferent vagotomies of the cervical vagus, rostral to the stimulation, in order to achieve significant inflammatory attenuation [1-5].

Of interesting note is that there seemed to be artificially lower levels of IL-6, IL-10, and IFN-γ in all cervical subgroups compared to the SubD Sham animals. This could indicate an inflammatory effect of the subdiaphragmatic surgery itself.

S6 Table 1 shows us very little nominal difference in the responses of GM-CSF, IL-22, and IL-17F, between CVes and CVns subsets.

**S4 Table 1. Fractional Analysis of Cytokine Cascades Over Given Threshold Concentrations for Cervical Subsets**

| **Cytokine** | **GM-CSF** | **IL-17F** | **IL-22** |
| --- | --- | --- | --- |
| Threshold | 80 pg/ml | 100 pg/ml | 100 pg/ml |
| **Subset** | | | |
| CVes | 1/5 | 1/5 | 1/5 |
| CVns | 1/3 | 1/3 | 2/3 |

*Fractions are the number of animals that had cytokine cascade elevations above the given threshold.*

*Colors are used for simplified interpretation and are not meant to signify results as desirable or not. Blue cells are subsets that had over half its samples rise above threshold. Yellow cells are subsets that had half or less of its samples rise above threshold.*

1. Borovikova LV. Vagus nerve stimulation attenuates the systemic inflammatory response to endotoxin. Nature. 2000;405(6785):458-63.

2. Huston JM, Ochani M, Rosas-Ballina M, Liao H, Ochani K, Pavlov VA, et al. Splenectomy inactivates the cholinergic antiinflammatory pathway during lethal endotoxemia and polymicrobial sepsis. J Exp Med. 2006;203(7):1623-8.

3. Rosas-Ballina M, Ochani M, Parrish WR, Ochani K, Harris YT, Huston JM, et al. Splenic nerve is required for cholinergic antiinflammatory pathway control of TNF in endotoxemia. Proceedings of the National Academy of Sciences of the United States of America. 2008;105(31):11008. doi: 10.1073/pnas.0803237105.

4. Bratton BO, Martelli D, McKinley MJ, Trevaks D, Anderson CR, McAllen RM. Neural regulation of inflammation: no neural connection from the vagus to splenic sympathetic neurons. Experimental Physiology. 2012;97(11):1180-5. doi: 10.1113/expphysiol.2011.061531.

5. Patel YA, Saxena T, Bellamkonda RV, Butera RJ. Kilohertz frequency nerve block enhances anti-inflammatory effects of vagus nerve stimulation. Scientific Reports. 2017;7:39810. doi: 10.1038/srep39810.
